# Isomeric 1,4‐Dihydropentalene‐Containing Building Blocks for High Mobility Ladder‐Type Conjugated Polymers

**DOI:** 10.1002/anie.202500860

**Published:** 2025-03-10

**Authors:** Shengnan Zhang, Yuqian Liu, Hao Dong, Yanru Li, Kai Zhang, Kaihu Xian, Yang Han, Long Ye, Martin Heeney, Zhuping Fei

**Affiliations:** ^1^ Institute of Molecular Plus Department of Chemistry and Haihe Laboratory of Sustainable Chemical Transformations Key Laboratory of Organic Integrated Circuits Ministry of Education Tianjin University Tianjin 300072 China; ^2^ School of Materials Science & Engineering Tianjin Key Laboratory of Molecular Optoelectronic Sciences and Collaborative Innovation Center of Chemical Science and Engineering Tianjin University Tianjin 300350 China; ^3^ Institute for Electric Light Sources School of Information Science and Technology Fudan University Shanghai 200433 China; ^4^ Division of Physical Sciences & Engineering Chemistry Program King Abdullah University of Science and Technology (KAUST) Thuwal 23955‐6900 Saudi Arabia

**Keywords:** Conjugated polymers, High mobility, Isomerization effect, Novel building blocks, Organic field‐effect transistors

## Abstract

Nearly amorphous conjugated polymers, though lacking long‐range order, can exhibit high charge carrier mobility and enhanced flexibility, making them ideal for flexible or stretchable OFET applications. However, research on novel structural blocks for this class of materials remains limited. We report the synthesis of two derivatives of the 5,10‐dihydroindeno[2,1‐*a*]indene (DHI) core, where two thieno groups are appended to each end to reduce torsional disorder with adjacent comonomers. The two 1,4‐dihydropentalene containing isomers, anti‐C16DHIT‐Br and syn‐C16DHIT‐Br, feature distinct thieno group orientations, leading to differences in their electronic properties. The anti‐isomer shows a fully delocalized conjugation pathway, whereas the syn isomer exhibits cross‐conjugation, limiting delocalization along the polymer backbone. These monomers were copolymerized with benzothiadiazole (BT) to form two conjugated polymers, anti‐C16DHIT‐BT and syn‐C16DHIT‐BT, both with highly planar backbones, good solubility, and solution processability. Morphological studies reveal that anti‐C16DHIT‐BT exhibits more ordered stacking and higher crystallinity than syn‐C16DHIT‐BT. Devices based on anti‐C16DHIT‐BT show significantly better performance, with a hole mobility of 2.38 cm^2^ V^−1^ s^−1^, slightly higher than the widely studied pIDTBT under identical conditions.

## Graphical abstract

We report the synthesis of two novel isomeric derivatives of 5,10‐dihydroindeno[2,1‐*a*]indeneo (DHI), incorporating a thieno group to reduce torsional disorder. The anti‐C16DHIT and syn‐C16DHIT isomers differ in conjugation characteristics, with the anti‐isomer showing fully delocalized conjugation, whereas syn isomer exhibiting cross‐conjugation. The polymer based on anti‐C16DHIT exhibits high hole mobility of 2.38 cm^2^ V^−1^ s^−1^, surpassing that of pIDTBT in same device structure.

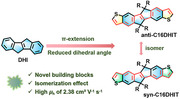

## Introduction

Conjugated polymers are a promising platform for the development of new generations of electronic devices. Their intrinsic synthetic tunability coupled with the good mechanical properties and potential for low cost, solution‐based fabrication has prompted much interest. For charge transporting applications, recent attention has focused on so‐called near‐amorphous conjugated polymers.^[^ ^1^, ^2^, ^3^ ^]^ Such materials lack the long range order associated with earlier classes of conjugated polymers, such as poly(3‐hexyl)thiophene (P3HT) or poly(2,5‐bis(3‐hexadecylthiophen‐2‐yl)thieno[3,2‐*b*]thiophene) (pBTTT), but nevertheless achieve high charge carrier mobility. They are particularly attractive for applications in flexible or stretchable electronics, where the higher degree of amorphous character can help accommodate mechanical changes. Among such polymers materials, pIDTBT {poly(indacenodithiophene‐co‐benzothiadiazole)} has been one of the most investigated.^[^ ^4^, ^5^ ^]^ High molecular weight pIDTBT with hexadecyl sidechains (pIDTBT‐C16) has demonstrated some the highest performing field‐effect transistor (FET) devices, with mobility up to 3.6 cm^2^ V^−1^ s^−1^ and without any of the nonlinearities often observed in high mobility materials.^[^ ^6^ ^]^

The high mobility observed in pIDTBT, despite the lack of any obvious long range order by X‐ray crystallography,^[^ ^7^ ^]^ has been intriguing. Studies have highlighted that that pIDTBT exhibits a highly linear backbone, with good coplanarity between the adjacent aromatic comonomers, which helps to promote good intrachain charge transport.^[^ ^8^, ^9^ ^]^ Interchain charge transfer requires close proximity of the conjugated backbones and is typically associated with crystalline polymer domains. For pIDTBT, it appears that films consist of unusual mesh‐like domains, with significant short and medium range order.^[^ ^10^, ^11^, ^12^ ^]^ Such films exhibit almost perpendicular crossing points between chains, with close contacts between benzothiadiazole (BT) monomers^[^ ^13^, ^14^ ^]^ helping to promote a highly interconnected transport network.

**Figure 1:**
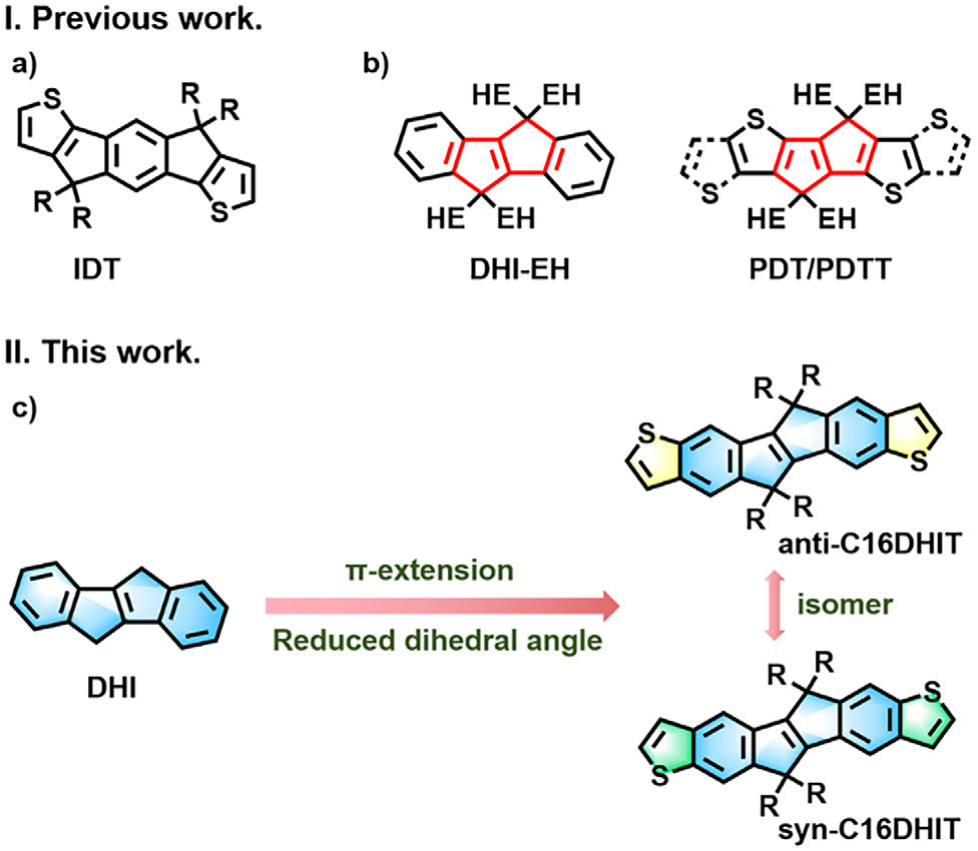
a) Structure of IDT core. b) Structures of existing multiple‐ring‐fused conjugated motifs containing 1,4‐dihydropentalene unit. c) Molecular design strategy and chemical structures of anti‐C16DHIT and syn‐C16DHIT in this work. (R, hexadecyl, EH, 2‐ethylhexyl).

The chemical structure of pIDTBT consists of a donor–acceptor type arrangement, where the ladder‐like donor monomer of IDT bears four solubilizing sidechains (Figure 1). The use of long and linear alkyl sidechains is important to promote high molecular weight and good device performance.^[^ ^15^ ^]^ The terminal thieno group of the IDT core is also important to facilitate good coplanarity with the adjacent BT monomer.^[^ ^9^, ^16^ ^]^ To improve on the performance of pIDTBT, various strategies have been attempted. Copolymerization of IDT with different comonomers has been extensively investigated,^[^ ^17^, ^18^, ^19^ ^]^ although BT derivatives remain some of the best performing. Changing the heteroatom in the IDT core from sulfur to selenium (pIDSeBT)^[^ ^20^ ^]^ has successfully improved performance, whereas changing the bridging atom from carbon to germanium or silicon reduces charge carrier mobility.^[^ ^21^, ^22^ ^]^

Increasing the number of fused aromatics in the donor core is also an important strategy. This primarily aims to improve intrachain transport and modify the polymer energy levels, but it also changes the density of the alkyl groups, potentially allowing more space for interchain crossing points.^[^ ^23^ ^]^ Thus, appending another thiophene ring to the each end of IDT (pIDTT‐BT)^[^ ^24^ ^]^ or replacing the thieno group with benzothiophene (forming dithiopheneindenofluorene, pTIF‐BT)^[^ ^25^ ^]^ resulted in improved transistor performance. Interestingly, lengthening the donor by increasing the number of fused aromatics in the donor core did not result in further improvements, with a reduction in mobility observed.^[^ ^26^ ^]^ This was rationalized by a reduction in efficient sidechain interdigitation leading to void spaces and increased film disorder.

In the studies above, the central portion of the IDT core (i.e., the “indaceno” part) was kept unchanged. In this arrangement, a central benzene ring separates the two fused 5‐membered rings that serve as a point of attachment for the required solubilizing groups. Given that the aromatic stabilization energy of this central benzene ring has a significant impact on the overall polymer energetics, it is desirable to explore other motifs. This has prompted the replacement of the benzene ring with naphthalene, which resulted in a significant reduction in FET performance^[^ ^27^ ^]^ or thieno[3,2‐*b*]thiophene^[^ ^28^ ^]^ in the related area of nonfullerene acceptors. We were interested in removing the aromatic ring altogether and having a bridged vinylene bond to promote polymer delocalization. It is also potentially attractive as it results in moving the sidechains further from the ends of the ladder monomer, potentially creating more space for the important crossing points between polymer chains. This strategy has been much less explored, probably due to synthetic challenge of incorporating the required central 1,4‐dihydropentalene system.^[^ ^29^ ^]^ The best explored class of polymers is based on 5,10‐dihydroindeno[2,1‐*a*]indene (DHI), in which two terminal benzene rings are linked to the central 1,4‐dihydropentalene (Figure 1). Polymers based on DHI exhibit excellent oxidative stability and have been explored for light‐emitting and photovoltaic applications.^[^ ^30^, ^31^, ^32^ ^]^ Zhu et. al. reported thiophene or thienothiophene flanked 1,4‐dihydropentalene derivatives, PDT and PDTT.^[^ ^29^ ^]^ These structures were utilized in the design of small molecule acceptors for high‐performance organic photovoltaics and photodetectors (Figure 1). However, to the best of our knowledge there are no examples reported in which the aromatic ends of the indenoindene core has been expanded.

Here, we report the synthesis of two novel derivatives of the DHI core, in which two thieno groups are appended onto either end. The thieno group is deliberately chosen to reduce torsional disorder with adjacent comonomers. We report two isomers of our new DHIT core, which differ with the relative geometry of the thieno groups (Figure 1). The two isomers differ in their ability to fully delocalize from one end of the core to the other, anti‐C16DHIT features a fully delocalized pathway, whereas syn‐C16DHIT exhibits cross‐conjugation and is not able to fully delocalize along the polymer backbone (see Figure S1 for a schematic). Such cross‐conjugated polymers are less explored in organic electronic applications but offer intriguing opportunities to further tune optoelectronic properties.^[^ ^33^ ^]^ Here, we report the copolymerization of both isomers with BT, and compare and contrast the properties of both polymers. We find that the anticopolymer exhibits significantly high charge carrier mobility, with performance comparable to pIBT‐BT benchmarked under the same device configuration.

## Results and Discussion

### Synthesis and Characterization

**Scheme 1:**
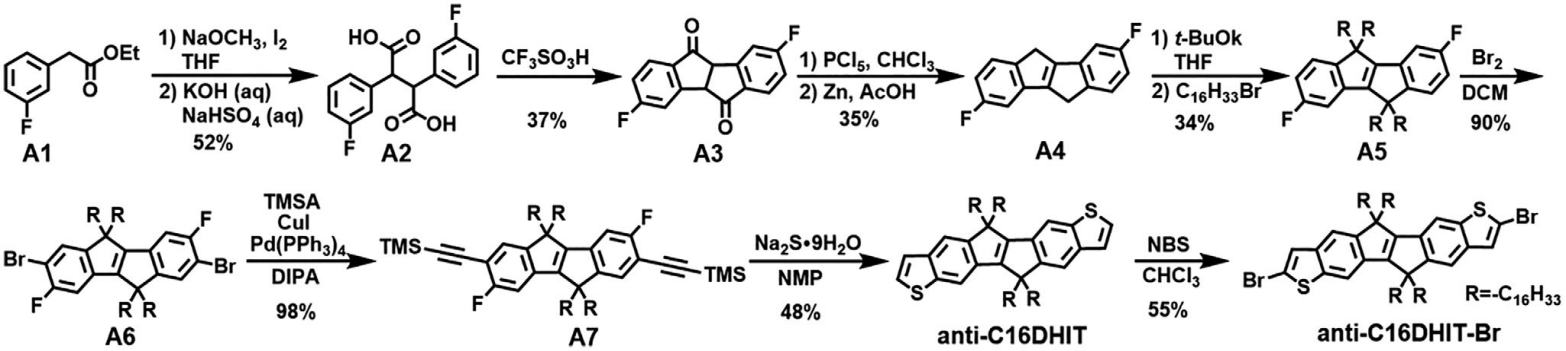
Synthesis route of the monomer anti‐C16DHIT‐Br.

**Scheme 2:**
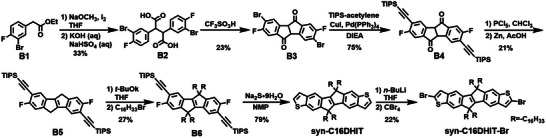
Synthesis route of the monomer syn‐C16DHIT‐Br.

**Scheme 3:**
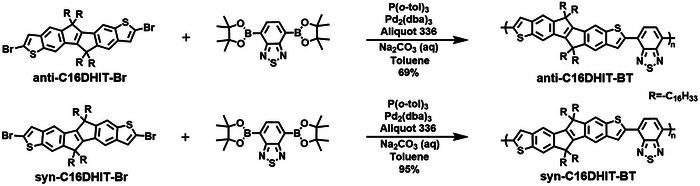
Synthesis route of the polymers.

The general synthetic routes of monomers anti‐C16DHIT‐Br, syn‐C16DHIT‐Br, and their respective polymers are shown in Schemes 1, 2, 3, respectively. The route builds on the reported preparation of 5,10‐dihydroindeno[2,1‐*a*]indene^[^ ^30^, ^34^ ^]^ to prepare previously unreported fluorinated derivatives, which were subsequently converted to the extended six ring species. The synthesis of anti‐C16DHIT‐Br was started from commercially available ethyl 2‐(3‐fluorophenyl)acetate (**A1**). In the presence of NaOCH_3_ and I_2_, **A1** undergoes a self‐coupling reaction, and the resulting intermediate is subsequently hydrolyzed under alkaline conditions to give **A2** in a yield of 52%.^[^ ^30^ ^]^ Then, **A3** was obtained by trifluoromethanesulfonic acid catalyzed Friedel–Crafts cyclisation reaction of **A2** in 37% yield. Dehydrogenation of the central ring together with reduction of the ketone groups was achieved by treatment with PCl_5_, following by reduction with Zn powder to afford the important intermediate **A4** in a yield of 35%. Subsequent alkylation of **A4** using *t‐*BuOK as base was performed in THF to afford desired **A5** in 34% yield.^[^ ^30^ ^]^ The terminal thieno groups were successfully added in three steps from **A5**. Initially, **A5** was brominated with elemental bromine to give **A6**, followed by a Sonogashira reaction to afford **A7**. Finally, nucleophilic substitution of the fluorine groups by Na_2_S·9H_2_O followed by in situ cyclisation of the resulting thiols to give anti‐C16DHIT, with a yield of 42% over three steps. Finally, the target monomer anti‐C16DHIT‐Br was obtained by bromination of anti‐C16DHIT with *N*‐bromosuccinimide (NBS) in a yield of 55%.

The isomeric monomer syn‐C16DHIT‐Br was synthesized from commercially available ethyl 2‐(3‐bromo‐4‐fluorophenyl)acetate (**B1**) (Scheme 2). In this case, the bromo groups required for the installation of the terminal thieno groups were present from the starting material to avoid later issues of regioselective bromination. Thus, using the same route as for the preparation of **A3**, **B3** was successfully prepared from **B1**, albeit in relatively low yields. **B3** was further converted to **B4** via a Sonogashira reaction in a yield of 75%. Under the same reaction conditions for the preparation of **A4**, **B5** was obtained in 21% yield. It is worth noting that the Sonogashira reaction step must be performed before the reduction reaction step in the process to prepare **B5** from **B3** as Br atoms could be eliminated under the reduction conditions used. Then, syn‐C16DHIT was smoothly prepared via alkylation and cyclization steps from **B5**. However, bromination of syn‐C16DHIT using same conditions to prepare anti‐C16DHIT‐Br gave a complex mixture, possibly due to the similar reactivities of multiple positions on syn‐C16DHIT. After optimization of the reaction conditions, syn‐C16DHIT‐Br was successfully prepared by deprotonation of the α‐position of the thiophene ring on syn‐C16DHIT, followed by the addition of CBr_4_.

Finally, anti‐C16DHIT‐BT and syn‐C16DHIT‐BT were prepared by copolymerization of anti‐C16DHIT‐Br or syn‐C16DHIT‐Br with 4,7‐bis(4,4,5,5‐tetramethyl‐1,3,2‐dioxaborolan‐2‐yl)benzo[*c*][1,2,5]thiadiazole via palladium‐catalyzed Suzuki coupling reactions (Scheme 3). Both polymers were purified by sequential Soxhlet extraction through methanol, acetone, hexane, and chloroform to remove low molecular weight oligomers and catalyst residues. The polymers showed excellent solubility in conventional organic solvents, such as tetrahydrofuran, chloroform, chlorobenzene, and so forth, due to the introduction of long linear alkyl side chains. The number average molecular weight (*M*_n_) and dispersity (*Ð*) of the polymers were determined by high‐temperature gel permeation chromatography (GPC) using a polystyrene standard calibration, and the data is summarized in Table 1. The *M*_n_/*Ð* of anti‐C16DHIT‐BT and syn‐C16DHIT‐BT were 33 KDa/2.25 and 34 kDa/1.72, respectively. The similarity in molecular weights and distributions enables a direct comparison between the two polymers.

**Table 1:** Physical, optical, and electrochemical properties of the polymers studied in this paper.

Polymer	*M*_n_ (KDa)	*Đ*	*T*_d_ (°C)	*λ*_abs max_^sol^ (nm)	*λ*_abs max_^film^ (nm)	*λ*_onset_ (nm)	*E*_g_^opt^ ^a)^ (eV)	HOMO^b)^ (eV)	LUMO^c)^ (eV)
anti‐C16DHIT‐BT	33	2.25	431	600	617	651	1.91	−5.46	−3.55
syn‐C16DHIT‐BT	34	1.72	425	517	538	569	2.18	−5.67	−3.49

^a)^ Optical bandgap estimated from the low energy band edge in the optical spectrum (*E*_g_^opt^ = 1240/*λ*_onset_).

^b)^ HOMO level estimated from cyclic voltammetry (CV).

^c)^ LUMO = HOMO + *E*_g_^opt^.

### Thermal Properties

The thermal properties of the polymers were investigated using thermogravimetric analysis (TGA) and differential scanning calorimetry (DSC). All polymers showed excellent thermal stability with decomposition temperatures (*T*_d_) at a 5% loss (*T*_d_) around 425 °C (Figure S2), which can meet the requirements for subsequent device fabrication. The polymers showed no obvious thermal transitions in the range of 30–300 °C (Figure S3), which is a common phenomenon for rigid rod conjugated polymers.

### Theoretical Calculations

**Figure 2:**
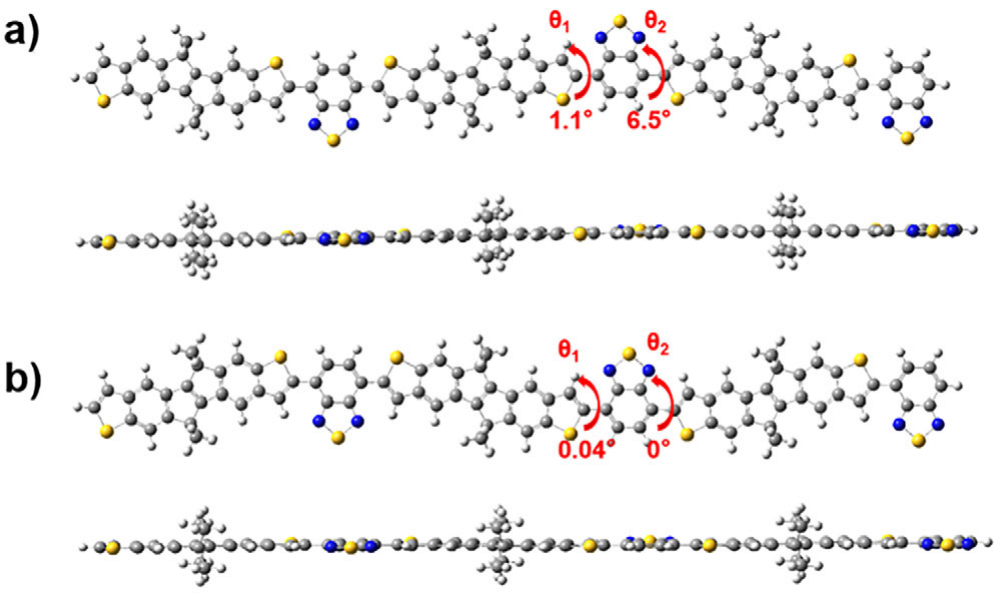
Top and side view of energy‐minimized structures (B3LYP‐D3(BJ)/6‐31G*) of a methyl‐substituted trimer of a) anti‐C16DHIT‐BT and b) syn‐C16DHIT‐BT.

Density functional theory (DFT) calculations of trimers of repeat units of polymers (Figure S4) were performed using a Gaussian model at the B3LYP‐D3(BJ)/6‐31G* level to study the energy levels, optimized backbone conformations, and charge distributions of polymers, where the side chains were substituted with methyl groups to simplify the calculations. The highest occupied molecular orbitals (HOMO) and the lowest unoccupied moecular orbitals (LUMO) energy levels were calculated under PBE0/Def2TZVP level. As shown in Figure 2, both polymers exhibit a near‐planar geometry with very small dihedral angles between different units of backbone (Table S1), which can potentially favor molecular assembly and carrier transport properties of the materials. The HOMOs of anti‐C16DHIT‐BT and syn‐C16DHIT‐BT are distributed over the entire length of the conjugation backbone, whereas the LUMOs are mainly located in the electron‐deficient benzothiodiazole units (Figure S5). These frontier molecular orbitals (FMO) distributions is similar to those of other donor–acceptor analogues, such as IDTTBT and IDSeBT.^[^ ^20^, ^24^ ^]^ From the perspective of conjugation, the backbone of anti‐C16DHIT‐BT exhibits a linear conjugation pattern, whereas the backbone of syn‐C16DHIT‐BT displays a cross‐conjugation configuration (Figure S6). The predicted HOMO–LUMO energy levels of anti‐C16DHIT‐BT and syn‐C16DHIT‐BT are −5.17/−2.97 eV and −5.44/−2.90 eV, respectively. Compared to anti‐C16DHIT‐BT, syn‐C16DHIT‐BT has a shallower HOMO level and smaller band gap (2.00 eV vs. 2.54 eV). This is consistent with reports in the literature that polymers with cross‐conjugation configuration tends to have larger bandgaps.^[^ ^20^, ^35^ ^]^

### Optical and Electronic Properties

**Figure 3:**
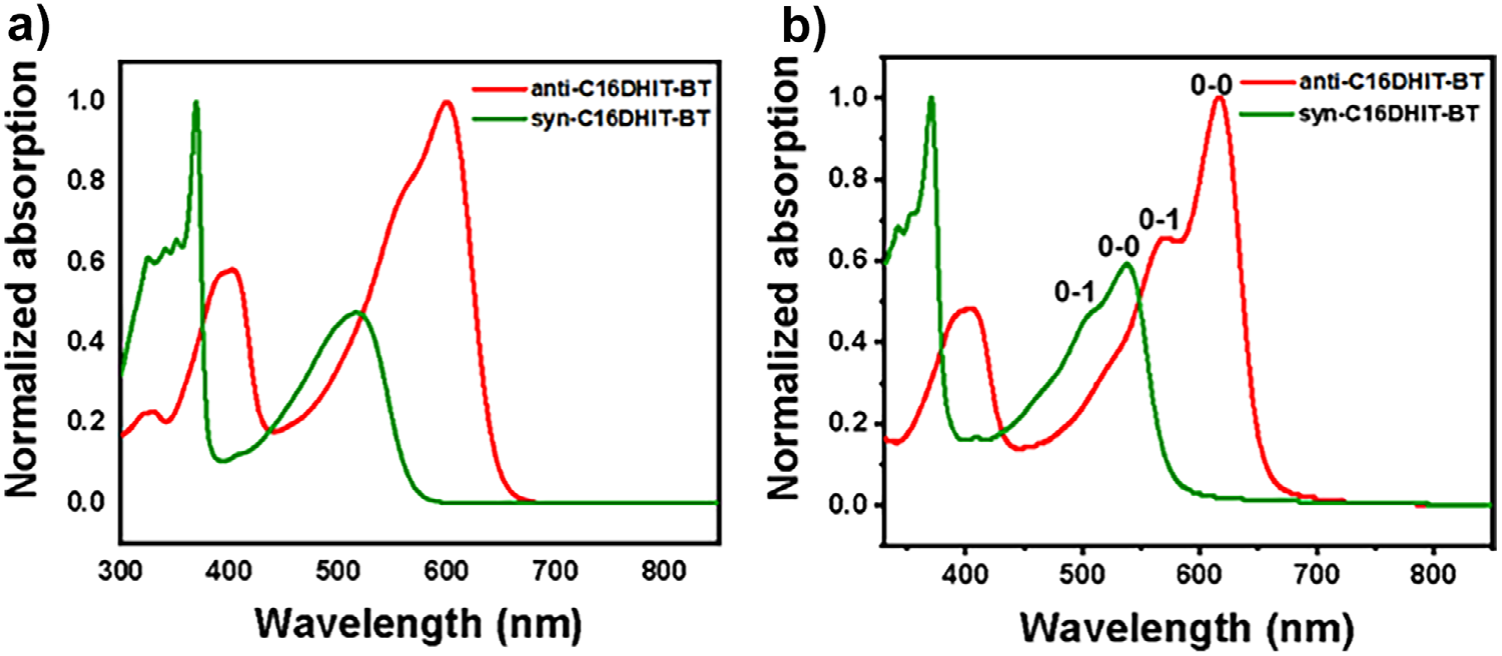
Normalized UV–vis–NIR absorption spectra of the polymers in a) chlorobenzene solution and b) in film.

The UV–vis absorption spectra of the polymers in chlorobenzene solution and in thin films are shown in Figure 3, and the corresponding data are summarized in Table 1. Two distinct absorption bands were observed for both polymers in dilute chlorobenzene solutions, where the higher energy absorption band was associated with the π–π* transitions of the conjugated backbone, whereas the longer wavelength bands were ascribed to the intramolecular charge transfer (ICT) from the DHIT units to benzothiadiazole units. The absorption spectra of the thin films of both polymers exhibited a redshift around 20 nm and an increase in the intensity of their 0–0/0–1 peaks versus the π–π* transitions compared to those of the solution state, which is attributed to stronger aggregation due to increased intermolecular interactions in the solid state. The maximum absorption peak (*λ*_max_ = 617 nm) of anti‐C16DHIT‐BT in the film was significantly red‐shifted compared to that of syn‐C16DHIT‐BT (*λ*_max_ = 538 nm). This also can be ascribed to the cross‐conjugation configuration of syn‐C16DHIT‐BT that acts to reduce effective delocalization.^[^ ^33^, ^36^ ^]^ The optical bandgaps of anti‐C16DHIT‐BT and syn‐C16DHIT‐BT in the solid state were calculated to 1.91 and 2.18 eV, respectively.

The electrochemical properties of the drop‐cast polymer films on a glassy carbon electrode were evaluated by cyclic voltammetry (CV). The electrochemical curves of polymers was shown in Figure S7, and the data are summarized in Table 1. The HOMO energy levels of anti‐C16DHIT‐BT and syn‐C16DHIT‐BT were calculated based on the onset oxidation potential as −5.46 and −5.67 eV, respectively. Reduction potentials could not be clearly observed from the CV, so the LUMO energy levels were estimated to be −3.55 and −3.49 eV by the additional of the optical bandgap. Anti‐C16DHIT‐BT had the smaller bandgap and deeper HOMO level than those of syn‐C16DHIT‐BT, which is in agreement with the trend of DFT calculations.

### Thin‐Film Transistor Properties

**Figure 4:**
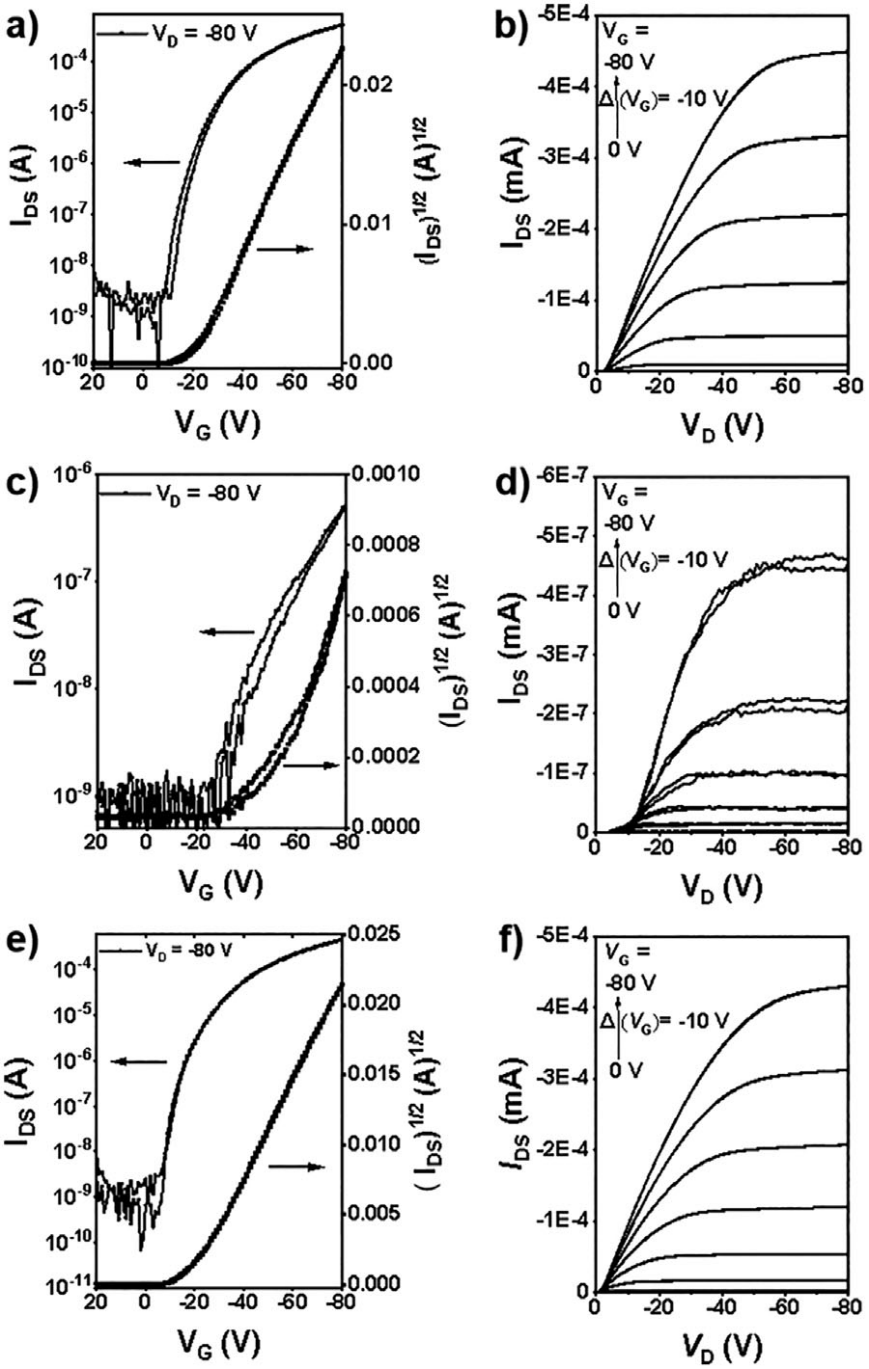
Representative transfer and output characteristics of OFET devices based on a,b) anti‐C16DHIT‐BT, c,d) syn‐C16DHIT‐BT, and e,f) pIDTBT.

Organic thin‐film transistors (OFETs) with bottom‐contact/top‐gate (BC/TG) structures were fabricated to investigate the charge‐transport properties of the polymers. Prepatterned gold source‐drain (S‐D) electrodes were modified with pentafluorothiophenol (PFBT) to facilitate hole injection.^[^ ^37^, ^38^ ^]^ The transfer and output characteristic curves (Figure 4) of the polymers were tested under ambient conditions, and the data is summarized in Table 2. Due to the deep HOMO levels and high LUMO levels, both polymers exhibited typical p‐type transport behavior. Representative transfer and output characteristic curves of anti‐C16DHIT‐BT exhibited negligible hysteresis and good drain current saturation, showing high average and maximum mobilities of 2.20 and 2.38 cm^2^ V^−1^ s^−1^, with on–off ratio of 10^4^–10^5^, respectively. In contrast, syn‐C16DHIT‐BT exhibits significantly inferior device performance, with low average mobility of 0.008 cm^2^ V^−1^ s^−1^, small on–off ratio of 10^2^–10^3^ and high current leakage. These features can be partially attributed to the cross‐conjugation configuration of polymer backbone, as well as the charge injection barriers resulting from the bigger energy level difference between its low HOMO level and the work function of the metal electrodes.

**Table 2:** OFET performance parameters based on anti‐C16DHIT‐BT, syn‐C16DHIT‐BT, and pIDTBT.

Polymer	*μ*_h,sat ave_ (*μ*_h,sat max_) [cm^2^ V^−1^ s^−1^]	*V*_T_ [V]	*I*_on_/*I*_off_
anti‐C16DHIT‐BT	2.20 ± 0.19 (2.38)	−20.0 ± 1.6	10^4^–10^5^
syn‐C16DHIT‐BT	0.008 ± 0.0042 (0.014)	−42.4 ± 5.9	10^2^–10^3^
pIDTBT	1.67 ± 0.17 (1.78)	−17.6 ± 3.1	10^4^–10^5^

To further evaluate the device performance of anti‐C16DHIT‐BT, an identical device using the well‐known ladder polymer pIDTBT of similar molecular weight was prepared by same procedure. The result showed that anti‐C16DHIT‐BT had a higher mobility compared to pIDTBT (average and maximum mobility of 1.67 and 1.79 cm^2^ V^−1^ s^−1^) (Figure 4), demonstrating that incorporating 1,4‐dihydropentalene into fused‐ring structures is a useful pathway for developing high‐mobility semiconductor materials in OFETs.

### Morphological Features and Microstructure

**Figure 5:**
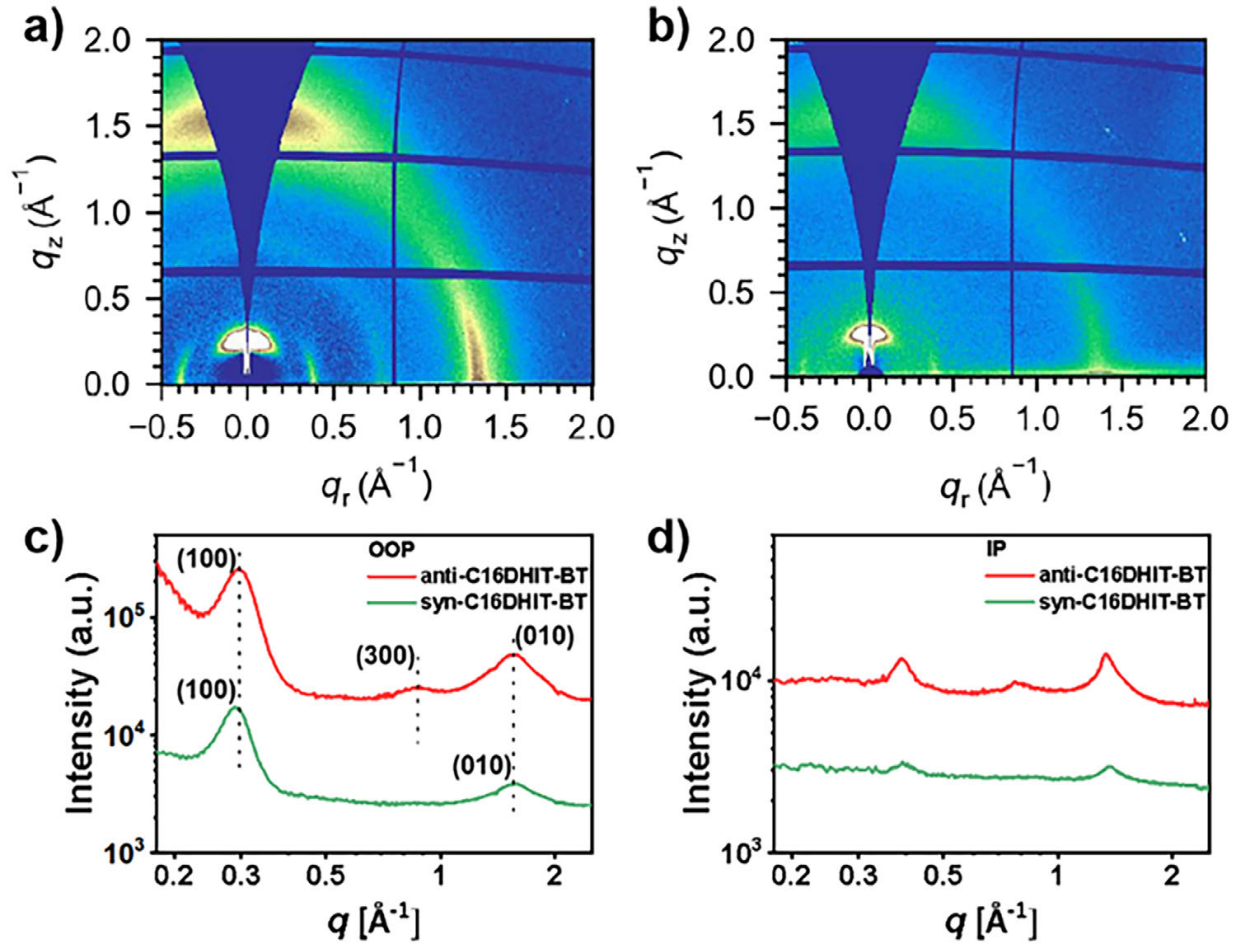
2D GIWAXS patterns of a) anti‐C16DHIT‐BT and b) syn‐C16DHIT‐BT. Corresponding c) out‐of‐plane and d) in‐plane sector‐averaged profiles for the polymer films.

The microstructure and orientation of the polymers at the molecular level were investigated using grazing‐incidence wide‐angle X‐ray scattering (GIWAXS). The corresponding 1D plots (out‐of‐plane, OOP; in‐plane, IP) and parameters are summarized in Figure 5 and Tables S2 and S3. In the OOP direction, anti‐C16DHIT‐BT film exhibited a peak (010) at a value of *q* = 1.56 Å^−1^, corresponding to a *d*‐spacing of 4.03 Å and suggesting a face‐on orientation. A peak at a similar distance has been assigned to π–π stacking for previously reported pIDTBT and pTIF‐BT with the same side chains.^[^ ^4^, ^5^, ^25^ ^]^ Corresponding IP peaks at *q* = 0.39 Å^−1^ and *q* = 0.81 Å^−1^ are also observed, which may result from either lamellar packing (i.e., (*n*00) peaks) or from periodicity along the backbone direction (00*l*) as observed in pIDTBT and pTIF‐BT. Examination of polymers DHIT‐BT with different sidechain lengths would help to fully assign these peaks. There also appears to be a population of edge‐on polymers, with corresponding *d*‐spacings in the OOP direction (Table S2), indicating that anti‐C16DHIT‐BT adopted a mixed face‐on and edge‐on packing orientation in the film, which results in efficient 3D charge transport channels to facilitate charge transport. Similarly to anti‐C16DHIT‐BT, syn‐C16DHIT‐BT also exhibited a mixed face‐on and edge‐on packing orientation in the film, with similar lamellar and π–π stacking distances. Comparing the two polymer films, anti‐C16DHIT‐BT demonstrates higher‐order lamellar diffraction peaks and more intense diffraction signals. This suggests that anti‐C16DHIT‐BT has a more ordered molecular packing and higher overall crystallinity, in agreement with the differences observed in device performance.

**Figure 6:**
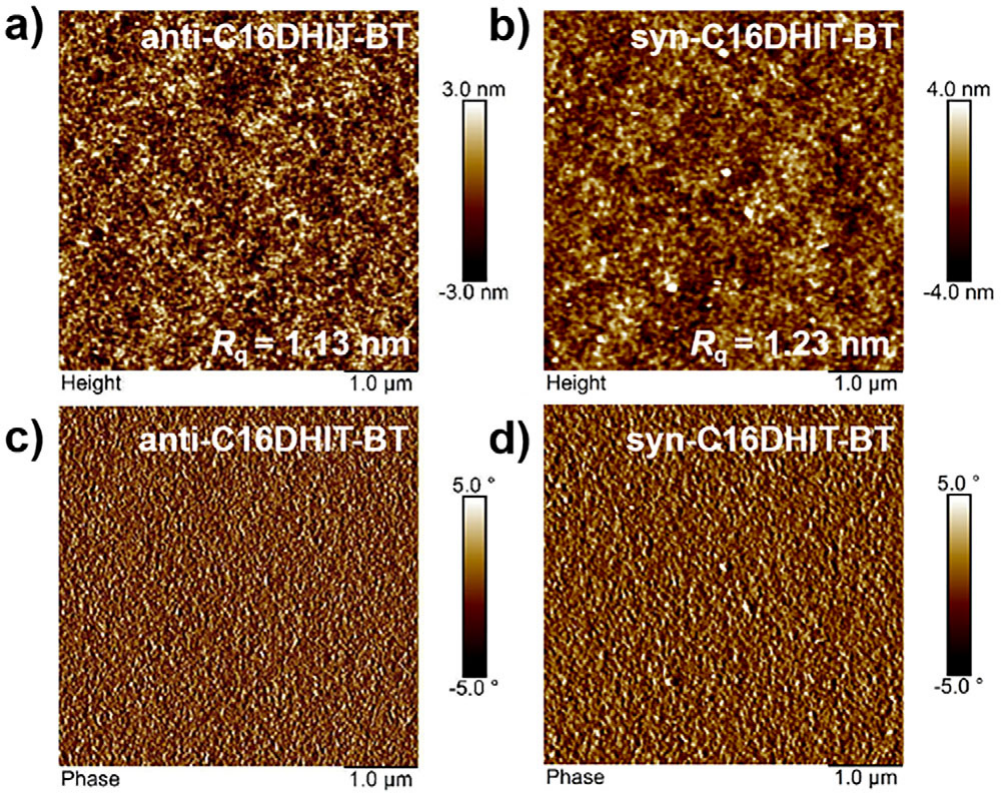
a,b) AFM topography images and c,d) phase images (5 µm × 5 µm) of a,c) anti‐C6DHIT‐BT and b–d) syn‐C6DHIT‐BT.

The topology of the polymer films was characterized by atomic force microscopy (AFM) (Figure 6). Both anti‐C16DHIT‐BT and syn‐C16DHIT‐BT films formed continuous smooth fiber‐like structures, with root‐mean‐square roughness (RMS) of 1.13 and 1.23 nm, respectively. The low interface roughness coupled with the continuous microstructure of films leads to a large degree of device uniformity and renders these polymers as promising materials for large‐area thin‐film electronic applications.

## Conclusion

In summary, through the design and optimization of synthetic routes, we successfully synthesized two isomeric donor monomers (anti‐C16DHIT‐Br and syn‐C16DHIT‐Br) incorporating a central 1,4‐dihydropentalene structure, which exhibit linear conjugation and cross‐conjugation characteristics, respectively. These monomers were copolymerized with BT to yield two conjugated polymers, namely, anti‐C16DHIT‐BT and syn‐C16DHIT‐BT. Both polymers feature highly planar backbone structures and exhibit good solubility and solution processability. Morphology and microstructure studies have shown that both polymer films exhibit low surface roughness and mixed face‐on and edge‐on stacking orientation. Anti‐C16DHIT‐BT displays more ordered layer stacking and higher crystallinity compared to syn‐C16DHIT‐BT. Together with the fact that anti‐C16DHIT‐BT has linear conjugation characteristics, whereas syn‐C16DHIT‐BT exhibits cross‐conjugation characteristics, devices based on anti‐C16DHIT‐BT demonstrated significantly better performance compared to those based on syn‐C16DHIT‐BT. Moreover, anti‐C16DHIT‐BT exhibited a high hole mobility of 2.38 cm^2^ V^−1^ s^−1^, comparable to the widely studied pIDTBT. Our research indicates that incorporating the dihydropentalene fragment into the design of conjugated polymers is an effective strategy for achieving high‐performance organic thin‐film transistor materials.

## Supporting Information

The Supporting Information comprises comprehensive characterization and test data for the synthesized compounds, including ^1^H, ^13^C, and ^19^F NMR spectra, MALDI‐TOF MS spectra, TGA graphs, DSC curves, AFM data, and 2D‐GIWAXS data, as well as the preparation methodology for OFETs. Furthermore, it offers detailed insights into the computational methods employed and the associated results. All pertinent data are included to substantiate the findings reported in the main manuscript.

## Conflict of Interests

The authors declare no conflict of interest.

## Supporting information



Supporting Information

## Data Availability

The data that support the findings of this study are available from the corresponding author upon reasonable request.
