# Supplementary material for: Isomeric 1,4‐Dihydropentalene‐Containing Building Blocks for High Mobility Ladder‐Type Conjugated Polymers
Source: Angew Chem Int Ed Engl. 2025 Mar 10;64(19):e202500860. doi: 10.1002/anie.202500860 (PMC12051780; doi:10.1002/anie.202500860)
Supplement: Supplementary file 1 — Supporting Information [file ANIE-64-e202500860-s001.docx]

Supporting Information

Isomeric 1,4-Dihydropentalene-containing Building Blocks for High Mobility Ladder-Type Conjugated Polymers

Shengnan Zhang,^#^ Yuqian Liu,^#^ Hao Dong, Yanru Li, Kai Zhang, Kaihu Xian, Yang Han,* Long Ye, Martin Heeney* and Zhuping Fei*

|  | **Page** |
| --- | --- |
| **Instruments and experimental conditions** | **S2** |
| **Fabrication and characterization of organic thin-film transistors (OFETs)** | **S3** |
| **Experimental section** | **S4-S10** |
| **Figure S1.** Schematic representation of the two connection modes in anti-C16DHIT and syn-C16DHIT. | **S11** |
| **Figure S2.** TGA curves of the polymers. | **S11** |
| **Figure S3.** DSC curves of the polymers. | **S12** |
| **Figure S4.** The chemical structure of trimers of the polymers. | **S12** |
| **Table S1.** Calculated dihedral angles and energy levels for minimum energy geometries of trimers. | **S12** |
| **Figure S5.** The frontier orbitals of a) anti-C16DHIT-BT and b) syn-C16DHIT-BT calculated by DFT. | **S13** |
| **Figure S6.** Schematic representation of the two connection modes in linear and cross-conjugated copolymers. | **S13** |
| **Figure S7.** Film cyclic voltammograms (CV) of the polymers. | **S14** |
| **Table S2.** Data summary of the edge-on oriented population of 2D GIWAXS patterns of the polymers. | **S14** |
| **Table S3.** Data summary of the face-on oriented population of 2D GIWAXS patterns of the polymers. | **S14** |
| **Figure S7-S51.** ^1^H NMR, ^13^C NMR, ^19^F NMR spectrum and MALDI-TOF-MS. | **S15-37** |

**Instruments and experimental conditions**

^1^H and ^13^C NMR spectra were measured on JNM-ECZ600R (600 MHz) and JNM-ECZ400R (400 MHz) with all chemical shifts given in parts per million (ppm). Matrix Assisted Laser Desorption Ionization (MALDI) mass spectra were performed on Bruker Daltonics UltrafleXtreme time of flight (TOF) equipment. GPC analysis was recorded on a PL-GPC 220 system using 1,2,4-trichlorobenzene as the eluent and polystyrene as the standard at 150 ℃. Thermogravimetric analysis (TGA) was performed on PerkinElmer Pyris 1 thermogravimetric analyzer under a nitrogen atmosphere at a heating rate of 10 ℃/min to record TGA curves. Differential scanning calorimetry (DSC) was carried out on a DSC214 Polyma at a ±10 ℃/min heating/cooling rate at a nitrogen flow. UV-vis-NIR absorption spectra were obtained by DAOJIN UV-19001 spectrophotometer. The solution spectra were measured with the concentration of 1×10^-5^ mol/L of the repeating units, and thin films were prepared by spin-coating CB solutions on quartz substrates (4 mg/mL). Absorption onset of films were used to calculated the optical bandgap (*E*_g_^opt^ = 1240/*λ*_onset_ eV). The electrochemical behavior of compounds was investigated by cyclic voltammetry (CV) (ShangHai ChenHua, CHI760E) with a standard three-electrode electrochemical cell in a 0.1 M *n*-Bu4NPF6 solution in CH_3_CN at room temperature at N_2_ atmosphere with a scanning rate of 50 mV·s^−1^. A glassy carbon electrode with 10 mm diameter, a Pt wire and a saturated calomel electrode were as the working, counter and reference electrodes, respectively. The CV film samples were prepared by drop-coating polymer solution in chlorobenzene (CB) on working electrode. The highest occupied molecular orbital (HOMO) and the lowest unoccupied molecular orbital (LUMO) energy levels were calculated using the following equations: HOMO = −(4.80 + *E*_onset_^ox^) eV and LUMO = −(4.80 + *E*_onset_^re^) eV, where *E*_onset_^ox^ and *E*_onset_^re^ were onset oxidation and reduction onset potentials of the polymers versus Fc/Fc^+^, respectively. Atomic force microscopy (AFM) images were recorded in tapping mode on a Bruker MutiMode 8 atomic force microscope.

**Fabrication and characterization of organic thin-film transistors (OFETs)**

Top-gate bottom-contact (TGBC) OFETs were fabricated on silicon wafers covered with a 300 nm SiO_2_ layer. Firstly, cleaning the substrates with deionized water, acetone and isopropanol, followed by dried at a nitrogen flow. Then heating the substrates to 120 °C for 30 min to remove solvent residues and cooling them under vacuum. Au used as source and drain electrodes (~32 nm) was firstly deposited in the bare substrate through a special shadow mask (W/L = 70, W = 5600 µm, L = 80 µm). And PFBT was used to treat the S-D electrode to facilitate charge injection. The polymer film was formed by spin-coating with a chlorobenzene solution (5 mg/mL), followed by thermal annnealing at 150 ℃ for 10 min. The CYTOP was then spin-coated as a dielectric layer at 2000 rpm for 120 s, followed by a gradient temperature increase in the range of 45-95 °C then annealed in N_2_ for 40 min. Thermal evaporation of the aluminum gate electrode (~80 nm) under vacuum conditions with shading was performed to obtain the final transistors. All measurements of the OFETs devices were performed on a Keysight B1500A analyzer. The saturation mobility was calculated as:

*I*_DS_ = (W/2L) *C*_i_ *μ* (*V*_G_ - *V*_T_)^2^

Where W/L is the channel width/length, *C*_i_ is the capacitance per unit area of gate dielectric layer, and *V*_G_ and *V*_T_ are the gate voltage and threshold voltage, respectively.

**Experimental section**

All chemicals and solvents were commercially purchased without further purification unless otherwise stated. *N*, *N*-Dimethylformamide (DMF) and chloroform (CHCl_3_) were distilled to dryness using CaH_2_. Tetrahydrofuran (THF), ether and toluene were distilled to dryness using sodium. All water and oxygen sensitive reactions were carried out under Ar atmosphere.

*2,3-bis(3-fluorophenyl)succinic acid (****A2****)*.

To a solution of sodium methoxide (4.80 g, 88.92 mmol) in THF (130 mL) was added ethyl 2-(3-fluorophenyl)acetate (**A1**) (16.20 g, 88.92 mmol) at -78 °C under argon, followed by the addition of a solution of iodine (11.28 g, 44.46 mmol) in THF (65 mL) over 10 min. The mixture was stirred at room temperature for 5 h before 5% aqueous sodium bisulfate solution (18 mL) was added. Then, the mixture was added potassium hydroxide (18.46 g, 329.00 mmol) in water (270 mL). After stirring at 40 °C overnight, the reaction mixture was treated with concentrated hydrochloric acid (15 mL). After cooling to room temperature, the precipitate was filtered, washed with 30 mL of water, and dried under reduced pressure at room temperature for 24 h to afford **A2** as white powder (7.07 g, yield 52%). ^1^H NMR (CDCl_3_, 600 MHz): *δ* (ppm) 7.16 (q, *J* = 7.9 Hz, 2H), 6.88 (t, *J* = 9.4 Hz, 6H), 4.25 (s, 2H); ^13^C NMR (CDCl_3_, 151 MHz): *δ* (ppm) 178.81, 163.61, 161.97, 136.28 (d, *J* = 7.4 Hz), 130.42 (d, *J* = 8.3 Hz), 124.20 (d, *J* = 2.6 Hz), 115.39 (d, *J* = 1.7 Hz), 115.24 (d, *J* = 3.5 Hz), 54.44; ^19^F NMR (CDCl_3_, 376 MHz) : *δ* (ppm) -110.09 – -113.40 (m).

*3,8-difluoro-4b,9b-dihydroindeno[2,1-a]indene-5,10-dione* *(****A3****)*.

Compound **A2** (19.30 g, 63.02 mmol) was placed in a dried reaction tube under argon. CF_3_SO_3_H (100 mL) was added into the reaction tube. Then the tube was sealed and the mixture was heated to 75 °C and stirred for 18 h. After cooling to room temperature, the mixture was poured into ice water (200 mL) under stirring, and sat. aq. Na_2_CO_3_ solution (100 mL) was added slowly. The precipitant was filtered, washed by water (100 mL) and dried under vacuum. The solid was purified by silica gel chromatography (eluent ethyl acetate : hexane = 1:7) to afford **A3** as light yellow solid (6.38 g, yield 37%). ^1^H NMR (CDCl_3_, 400 MHz): *δ* (ppm) 7.72 (dd, *J* = 8.5, 5.2 Hz, 2H), 7.56 (dd, *J* = 8.4, 2.2 Hz, 2H), 7.14 (td, *J* = 8.6, 2.3 Hz, 2H), 4.39 (s, 2H); ^13^C NMR (CDCl_3_, 101 MHz): *δ* (ppm) 198.99, 169.11, 166.54, 152.53 (d, *J* = 10.7 Hz), 131.12 (d, *J* = 1.6 Hz), 127.27 (d, *J* = 10.6 Hz), 117.70 (d, *J* = 23.7 Hz), 113.60 (d, *J* = 23.2 Hz), 52.51 (d, *J* = 1.7 Hz); ^19^F NMR (CDCl_3_, 376 MHz) : *δ* (ppm) -98.28 – -100.58 (m).

*3,8-difluoro-5,10-dihydroindeno[2,1-a]indene (****A4****)*.

To a solution of **A3** (14.30 g, 52.92 mmol) in chloroform (45 mL) at room temperature under argon was added PCl_5_ (23.14 g, 111.13 mmol), and the mixture was stirred at 50 °C for 30 h. The resulting solution was concentrated under reduced pressure at room temperature. Then, the mixture was diluted with boiling AcOH (220 mL), and Zn powder (66.05 g, 1.01 mol) was added in batches. After stirring at 120 °C for 4 h, the suspension was filtered hot and washed with boiling AcOH. The filtrate was concentrated under reduced pressure. And the residue was purified by silica gel chromatography (eluent hexane) to give **A4** as white solid (4.45 g, yield 35%). ^1^H NMR (CDCl_3_, 400 MHz): *δ* (ppm) 7.41 (dd, *J* = 8.2, 5.0 Hz, 2H), 7.10 (dd, *J* = 8.9, 2.4 Hz, 2H), 6.89 (ddd, *J* = 9.4, 8.2, 2.4 Hz, 2H), 3.57 (s, 4H); ^13^C NMR (CDCl_3_, 101 MHz): *δ* (ppm) 163.82, 161.41, 152.27 (d, *J* = 3.4 Hz), 142.83 (d, *J* = 9.5 Hz), 142.35 (d, *J* = 2.4 Hz), 125.45 (d, *J* = 9.2 Hz), 111.35 (d, *J* = 23.0 Hz), 106.75 (d, *J* = 23.6 Hz), 32.27; ^19^F NMR (CDCl_3_, 376 MHz) : *δ* (ppm) -116.35–-116.43 (m).

*3,8-difluoro-5,5,10,10-tetrahexadecyl-5,10-dihydroindeno[2,1-a]indene (****A5****)*.

To a solution of **A4** (1.96 g, 8.16 mmol) in THF (60 mL) was added *t*-BuOK (7.32 g, 65.27 mmol) under argon. After stirred at 50 °C for 2 h, 1-bromohexadecane (19.93 g, 65.27 mmol) was added dropwise, and the reaction was heated to reflux overnight. After cooling, the mixture was poured into ice water (100 mL), and the precipitate was filtered, washed with water (20 mL) and methanol (20 mL) in turn. The residue was purified by recrystallization with DCM to give **A5** as white solid (3.16 g, yield 34%). ^1^H NMR (CDCl_3_, 400 MHz): *δ* (ppm) 7.18 (dd, *J* = 8.2, 5.1 Hz, 2H), 6.96 (dd, *J* = 8.9, 2.4 Hz, 2H), 6.85 (ddd, *J* = 9.5, 8.3, 2.4 Hz, 2H), 2.06 – 1.86 (m, 8H), 1.33 – 0.94 (m, 104H), 0.91 – 0.82 (m, 12H), 0.81 – 0.68 (m, 4H), 0.62 – 0.48 (m, 4H); ^13^C NMR (CDCl_3_, 101 MHz): *δ* (ppm) 163.51, 161.10, 156.23 (d, *J* = 3.3 Hz), 150.90 (d, *J* = 2.4 Hz), 141.71 (d, *J* = 9.1 Hz), 122.57 (d, *J* = 9.3 Hz), 111.10 (d, *J* = 22.8 Hz), 106.55 (d, *J* = 23.2 Hz), 53.71, 38.50, 32.03, 30.22 – 29.16 (overlapping C), 24.48, 22.79, 14.23; ^19^F NMR (CDCl_3_, 376 MHz) : *δ* (ppm) -116.58 – -116.71 (m).

*2,7-dibromo-3,8-difluoro-5,5,10,10-tetrahexadecyl-5,10-dihydroindeno[2,1-a]indene (****A6****)*.

A solution of **A5** (3.16 g, 2.78 mmol) in CHCl_3_ (40 mL) was cooled to 0 °C, and Br_2_ (0.30 mL, 5.83 mmol) diluted in CHCl_3_ (2 mL) was added dropwise. The mixture was stirred at 0 °C and room temperature successively for 2 h each in the absence of light. Saturated Na_2_CO_3_ aqueous solution (10 mL) was added to quench the reaction, then poured into water (100 mL) and extracted three times with CHCl_3_ (50 mL). The combined organic phase was dried by Na_2_SO_4_, filtered, and concentrated under reduced pressure. The crude product was purified by recrystallization of DCM to obtain **A6** as a white solid (3.24 g, yield 90%). ^1^H NMR (CDCl_3_, 400 MHz): *δ* (ppm) 7.40 (d, *J* = 6.3 Hz, 2H), 7.02 (d, *J* = 8.4 Hz, 2H), 2.05 – 1.87 (m, 8H), 1.35 – 0.96 (m, 104H), 0.93 – 0.81 (m, 12H), 0.79 – 0.66 (m, 4H), 0.63 – 0.50 (m, 4H); ^13^C NMR (CDCl_3_, 101 MHz): *δ* (ppm) 159.52, 157.09, 155.83 (d, *J* = 2.6 Hz), 152.27 (d, *J* = 2.7 Hz), 140.49 (d, *J* = 8.1 Hz), 126.48, 107.37 (d, *J* = 24.2 Hz), 105.40 (d, *J* = 22.3 Hz), 54.21, 38.38, 32.03, 30.13 – 29.31 (overlapping C), 24.50, 22.79, 14.22; ^19^F NMR (CDCl_3_, 376 MHz) : *δ* (ppm) -109.36 – -109.45 (m).

*((3,8-difluoro-5,5,10,10-tetrahexadecyl-5,10-dihydroindeno[2,1-a]indene-2,7-diyl)bis (ethyne-2,1-diyl))bis(trimethylsilane) (****A7****)*.

To a solution of **A6** (3.30 g, 2.55 mmol), Pd(PPh_3_)_4_ (0.15 g, 0.13 mmol), and CuI (0.24 g, 0.13 mmol) in DIPA (92 mL) was added the trimethylsilane acetylene (3.24 mL, 22.92 mmol) under argon. The mixture was stirred at 60 °C overnight. The mixture was cooled to room temperature and concentrated under reduced pressure. Then methanol was added to get the precipitate, and then filtered, washed with methanol. The residue was purified by silica plug (PE) and recrystallization (DCM + MeOH) successively to give **A7** as yellow solid (3.32 g, yield 98%). ^1^H NMR (CDCl_3_, 400 MHz): *δ* (ppm) 7.29 (d, *J* = 6.4 Hz, 2H), 6.96 (d, *J* = 9.1 Hz, 2H), 2.04 – 1.87 (m, 8H), 1.35 – 0.83 (m, 116H), 0.75 – 0.62 (m, 4H), 0.62 – 0.48 (m, 4H), 0.33 – 0.25 (m, 18H); ^13^C NMR (CDCl_3_, 101 MHz): *δ* (ppm) 164.30, 161.82, 157.55 (d, *J* = 3.3 Hz), 150.66 (d, *J* = 2.7 Hz), 141.80 (d, *J* = 8.9 Hz), 126.15 (d, *J* = 2.0 Hz), 107.48 (d, *J* = 17.3 Hz), 106.65 (d, *J* = 23.2 Hz), 100.21 (d, *J* = 3.7 Hz), 99.41, 53.97, 38.50, 32.03, 30.36 – 29.35 (overlapping C), 24.55, 22.79, 14.22; ^19^F NMR (CDCl_3_, 376 MHz) : *δ* (ppm) -111.66 – -111.74 (m).

*Synthesis of* ***anti-C16DHIT***.

A mixture of Na_2_S·9H_2_O (0.91 g, 3.79 mmol) and NMP (30 mL) was stirred at room temperature for 15 min under argon, then **A7** (1.20 g, 0.90 mmol) was added and heated at 185 °C for 12 h. The mixture was cooled down and NH_4_Cl saturated solution (20 mL) was added to quench the reaction. The precipitate was collected by filtration and washed with water and methanol. And the residue was purified by rapid silica gel chromatography (eluent hexane) to give **anti-C16DHIT** as yellow solid (0.53 g, yield 48%). ^1^H NMR (CDCl_3_, 400 MHz): *δ* (ppm) 7.76 (s, 2H), 7.69 (s, 2H), 7.38 (d, *J* = 5.4 Hz, 2H), 7.35 (d, *J* = 5.1 Hz, 2H), 2.21 – 2.00 (m, 8H), 1.33 – 0.94 (m, 104H), 0.90 – 0.77 (m, 16H), 0.65 – 0.55 (m, 4H); ^13^C NMR (CDCl_3_, 101 MHz): *δ* (ppm) 155.42, 153.16, 138.70, 138.20, 137.13, 124.86, 124.26, 116.82, 112.37, 53.56, 39.34, 32.02, 30.37 – 29.37 (overlapping C), 24.63, 22.79, 14.22.

*Synthesis of* ***anti-C16DHIT-Br***.

To a solution of **anti-C16DHIT** (0.37 g, 0.30 mmol) in CHCl_3_ (18 mL) was added NBS (0.11 g, 0.62 mmol) in batches at 0 °C. The mixture was stirred at 0 °C for 30 min and at room temperature for 2 h in the absence of light. Then the mixture was poured into water (100 mL) and extracted three times with DCM (30 mL). The combined organic phase was dried by Na_2_SO_4_, filtered, and concentrated under reduced pressure. The crude product was purified by plug (PE) and recrystallization (acetone + THF) successively to give **anti-C16DHIT-Br** as yellow solid (0.23 g, yield 55%). ^1^H NMR (CDCl_3_, 400 MHz): *δ* (ppm) 7.60 (s, 2H), 7.56 (s, 2H), 7.32 (s, 2H), 2.18 – 1.97 (m, 8H), 1.34 – 0.92 (m, 104H), 0.92 – 0.72 (m, 16H), 0.64 – 0.48 (m, 4H); ^13^C NMR (CDCl_3_, 101 MHz): *δ* (ppm) 155.48, 153.47, 139.85, 137.94, 136.88, 126.96, 116.04, 113.20, 111.59, 53.74, 39.14, 32.02, 30.23 – 29.35 (overlapping C), 24.55, 22.79, 14.22. MALDI-TOF-MS: m/z 1370.801 (100%).

*2,3-bis(3-bromo-4-fluorophenyl)succinic acid (***B2***).*

**B2** was synthesized from **B1** (25.00 g, 95.75 mmol) and the synthetic procedure is similar as described for **A2**. Yield: 33%, white solid (7.33 g). ^1^H NMR (CDCl_3_, 600 MHz): *δ* (ppm) 7.37 (dd, *J* = 6.3, 2.2 Hz, 2H), 7.02 (ddd, *J* = 8.4, 4.5, 2.2 Hz, 2H), 6.97 (t, *J* = 8.3 Hz, 2H), 4.18 (s, 2H); ^13^C NMR (CDCl_3_, 151 MHz): *δ* (ppm) 178.79, 159.85, 158.20, 133.16, 131.03, 131.01, 129.17, 129.12, 117.20, 117.05, 109.98, 109.83, 53.60; ^19^F NMR (CDCl_3_, 565 MHz) : *δ* (ppm) -106.63.

*3,8-dibromo-2,7-difluoro-4b,9b-dihydroindeno[2,1-a]indene-5,10-dione* (**B3**).

**B3** was synthesized from **B2** (1.0 g, 2.15 mmol) and the synthetic procedure is similar as described for **A3**. Yield: 23%, white solid (0.21 g). ^1^H NMR (CDCl_3_, 600 MHz): *δ* (ppm) 8.14 (d, *J* = 5.7 Hz, 2H), 7.41 (d, *J* = 6.9 Hz, 2H), 4.39 (s, 2H); ^13^C NMR (CDCl_3_, 151 MHz): *δ* (ppm) 198.81 (d, *J* = 2.6 Hz), 160.96, 159.29, 145.17 (d, *J* = 2.9 Hz), 135.45 (d, *J* = 6.6 Hz), 131.78, 119.49 (d, *J* = 23.0 Hz), 111.45 (d, *J* = 23.5 Hz), 51.98; ^19^F NMR (CDCl_3_, 565 MHz) : *δ* (ppm) -103.82.

*2,7-difluoro-3,8-bis((triisopropylsilyl)ethynyl)-4b,9b-dihydroindeno[2,1-a]indene-5,10-dione* (**B4**).

**B4** was synthesized from **B3** (0.57 g, 1.36 mmol) and the synthetic procedure is similar as described for **A7**. It is worth noting that trimethylsilane acetylene has been replaced by (triisopropylsilyl) acetylene (2.78 mL, 12.20 mmol). Yield: 75%, white solid (0.30 g). ^1^H NMR (CDCl_3_, 600 MHz): *δ* (ppm) 7.96 (d, *J* = 5.8 Hz, 2H), 7.35 (d, *J* = 7.5 Hz, 2H), 1.16 – 1.10 (m, 42H); ^13^C NMR (CDCl_3_, 151 MHz): *δ* (ppm) 199.54 (d, *J* = 3.2 Hz), 164.72, 163.03, 144.23 (d, *J* = 3.0 Hz), 135.53 (d, *J* = 7.5 Hz), 131.50 (d, *J* = 1.5 Hz), 120.58 (d, *J* = 18.6 Hz), 110.70 (d, *J* = 22.4 Hz), 103.46 (d, *J* = 3.8 Hz), 98.79, 52.36, 18.79 – 18.52 (overlapping C), 11.43 – 11.08 (overlapping C); ^19^F NMR (CDCl_3_, 565 MHz) : *δ* (ppm) -107.46.

*((2,7-difluoro-5,10-dihydroindeno[2,1-a]indene-3,8-diyl)bis(ethyne-2,1-diyl))bis(triisopropylsilane)* (**B5**).

**B5** was synthesized from **B4** (0.30 g, 0.48 mmol) using a similar synthetic procedure to that described for **A4**. Yield: 21%, yellow solid (60.00 mg). ^1^H NMR (CDCl_3_, 600 MHz): *δ* (ppm) 7.41 (d, *J* = 6.3 Hz, 2H), 7.21 (d, *J* = 9.0 Hz, 2H), 3.58 (s, 4H), 1.16 – 1.15 (m, 42H); ^13^C NMR (CDCl_3_, 151 MHz): *δ* (ppm) 162.67, 161.01, 149.68 (dd, *J* = 4.3, 2.1 Hz), 149.39 (d, *J* = 8.8 Hz), 136.94 (d, *J* = 2.5 Hz), 123.08 (d, *J* = 1.7 Hz), 112.88 (d, *J* = 23.2 Hz), 110.18 (d, *J* = 17.5 Hz), 100.53, 96.11 (d, *J* = 4.3 Hz), 33.18 (d, *J* = 2.1 Hz), 18.91 – 18.61 (overlapping C), 11.57 – 11.20 (overlapping C); ^19^F NMR (CDCl_3_, 565 MHz) : *δ* (ppm) -113.51.

*((2,7-difluoro-5,5,10,10-tetrahexadecyl-5,10-dihydroindeno[2,1-a]indene-3,8-diyl)-bis(ethyne-2,1-diyl))bis(triisopropylsilane)* (**B6**).

**B6** was synthesized from **B5** (0.25 g, 0.42 mmol) using a similar synthetic procedure to that described for **A5**. Yield: 27%, yellow solid (0.17 g). ^1^H NMR (CDCl_3_, 600 MHz): *δ* (ppm) 7.23 (d, *J* = 6.2 Hz, 2H), 7.00 (d, *J* = 9.0 Hz, 2H), 2.07 – 2.00 (m, 4H), 1.94 – 1.86 (m, 4H), 1.33 – 0.98 (m, 188H), 0.91 – 0.84 (m, 12H), 0.77 – 0.69 (m, 4H), 0.54 – 0.47 (m, 4H); ^13^C NMR (CDCl_3_, 151 MHz): *δ* (ppm) 163.31, 161.64, 158.74, 135.82 (d, *J* = 2.3 Hz), 122.45, 110.35 (d, *J* = 22.8 Hz), 109.62 (d, *J* = 17.4 Hz), 100.73, 96.07 (d, *J* = 4.9 Hz), 54.68, 38.49, 32.03, 31.02 – 28.20 (overlapping C), 24.56, 22.79, 18.77, 14.22, 11.42; ^19^F NMR (CDCl_3_, 565 MHz) : *δ* (ppm) -112.19.

*Synthesis of* ***syn-C16DHIT***.

**Syn-C16DHIT** was synthesized from **B6** (718 mg, 0.50 mmol) and the synthetic procedure is similar to that described for **anti-C16DHIT**. Yield: 79%, yellow solid (460 mg). ^1^H NMR (CDCl_3_, 600 MHz): *δ* (ppm) 7.76 (s, 2H), 7.70 (s, 2H), 7.42 – 7.38 (m, 4H), 2.22 – 2.14 (m, 4H), 2.11 – 2.01 (m, 4H), 1.33 – 0.93 (m, 104H), 0.93 – 0.80 (m, 16H), 0.68 – 0.54 (m, 4H); ^13^C NMR (CDCl_3_, 151 MHz): *δ* (ppm) 154.71, 153.35, 138.64, 138.13, 137.01, 125.23, 124.17, 115.81, 113.29, 53.70, 39.33, 32.03, 30.68 – 28.55 (overlapping C), 24.66, 22.79, 14.22.

*Synthesis of* ***syn-C16DHIT-Br***.

*N*-BuLi (1.6 M in hexanes, 0.24 mL, 0.37 mmol) was added dropwise to a solution of **syn-C16DHIT** (90.00 mg, 0.074 mmol) in THF (10 mL) at 0 °C. The mixture was stirred at the same temperature for 1 h, and then a solution of CBr_4_ (98.33 mg, 0.30 mmol) in THF (2 mL) was added dropwise. After the mixture was stirred at room temperature overnight and then water was added to quench the reaction. the resultant mixture was extracted three times with DCM (20 mL). The combined organic phase was dried by NaSO_4_, filtered, and concentrated under reduced pressure. The crude product was purified by silica plug (PE) and recrystallization (acetone + THF) successively to give **syn-C16DHIT-Br** as white solid (0.22 g, yield 22%). ^1^H NMR (CDCl_3_, 600 MHz): *δ* (ppm) 7.61 (s, 2H), 7.55 (s, 2H), 7.35 (s, 2H), 2.17 – 2.10 (m, 4H), 2.05 – 1.98 (m, 4H), 1.31 – 0.94 (m, 104H), 0.90 – 0.85 (m, 12H), 0.83 – 0.75 (m, 4H), 0.61 – 0.52 (m, 4H); ^13^C NMR (CDCl_3_, 151 MHz): *δ* (ppm) 154.79, 153.29, 138.44, 138.17, 138.09, 126.87, 115.03, 113.78, 112.52, 53.92, 39.11, 32.02, 30.15, 29.90 – 29.39 (overlapping C), 24.58, 22.79, 14.22. MALDI-TOF-MS: m/z 1371.046 (100%).

*Synthesis of* ***anti-C16DHIT-BT***.

To a 25 mL reaction tube was added **anti-C16DHIT-Br** (247.48 mg, 0.18 mmol), 2,1,3-benzothiadiazole-4,7-bis(boronic acid pinacol ester) (BT-2Bpin) (70.01 mg, 0.18 mmol), Pd_2_(dba)_3_ (2.48 mg, 2.71×10^-3^ mmol), P(*o*-tol)_3_ (3.29 mg, 0.011mmol), and 3 drops of Aliquat 336. The tube was degassed three times with argon/vacuum cycling. Toluene (4.5 mL) and aqueous 1 M Na_2_CO_3_ (0.9 mL), which had been degassed in advance were added, and then argon was added after thorough degassing. The reaction tube was closed and placed in an oil bath at 80 °C, which was heated to 120 °C and stirred for two days. A degassed solution of phenyl borate (2.20 mg, 0.10 mmol) in toluene (3 mL) was added and stirred for 12 hours, followed by bromobenzene (0.10 mL) for 12 hours at the same temperature. The mixture was cooled down and poured into the solution of methanol (150 mL) and concentrated HCl (20 mL). The precipitant was filtered and purified by Soxhlet extraction with methanol, acetone, hexane, and chloroform. The chloroform solution was concentrated under reduced pressure and, then precipitate into methanol to give **anti-C16DHIT-BT** as dark solid (0.22 g, yield 69%). GPC: *M_n_* = 33 g/mol, *M_w_* = 74 g/mol, *Đ* = 2.25; *λ_max_* (film) = 617 nm. ^1^H NMR (CDCl_3_, 600 MHz): *δ* (ppm) 8.73 – 8.58 (broad), 8.02 – 7.92 (broad), 7.87 – 7.71 (broad), 2.30 – 2.00 (broad), 1.30 – 0.55 (broad).

*Synthesis of* ***syn-C16DHIT-BT***.

**Syn-C16DHIT-BT** was synthesized from **syn-C16DHIT-Br** (0.20 g, 0.15 mmol) and the synthetic procedure is similar as described for **anti-C16DHIT-BT**, but the polymerization time was three days. Yield: 95%, orange solid (190 mg, Soxhlet extraction by CHCl_3_). GPC: *M_n_* = 34 g/mol, *M_w_* = 59 g/mol, *Đ* = 1.72; *λ_max_* (film) = 538 nm. ^1^H NMR (CDCl_3_, 600 MHz): *δ* (ppm) 8.72 – 8.58 (broad), 8.07 – 7.93 (broad), 7.91 – 7.72 (broad), 2.49 – 1.91 (broad), 1.39 – 0.48 (broad).


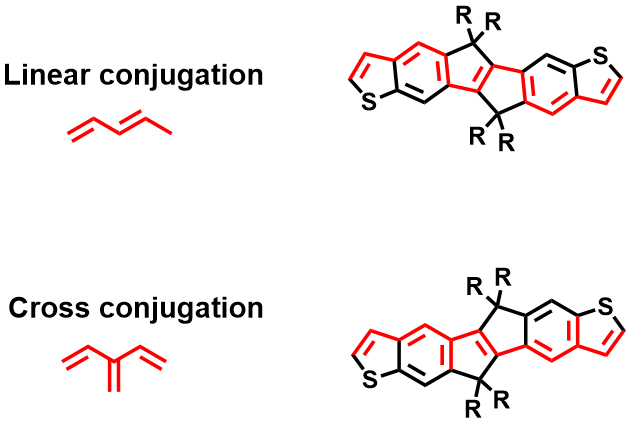


Figure S1. Schematic representation of the two connection modes in anti-C16DHIT and syn-C16DHIT.


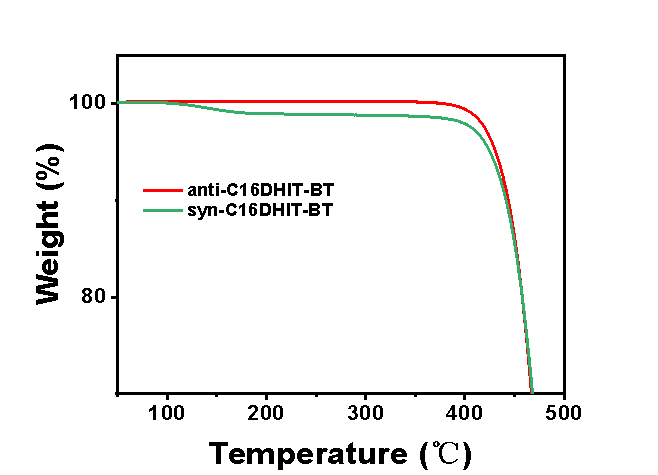


Figure S2. TGA curves of the polymers.


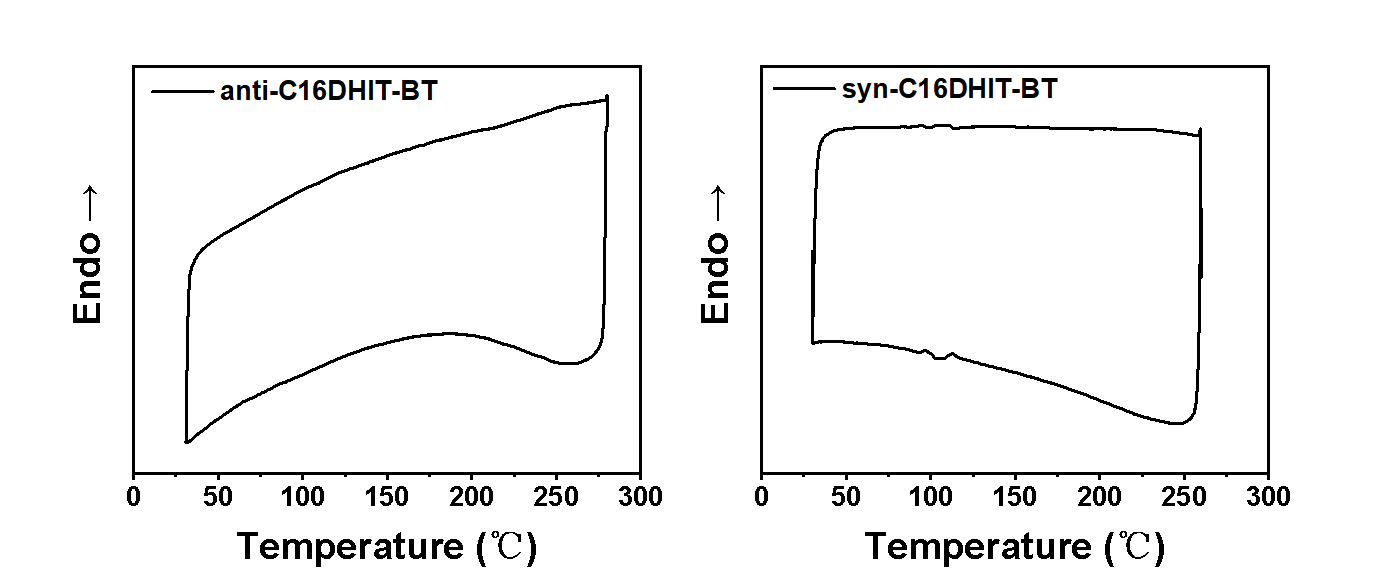


Figure S3. DSC curves of the polymers.


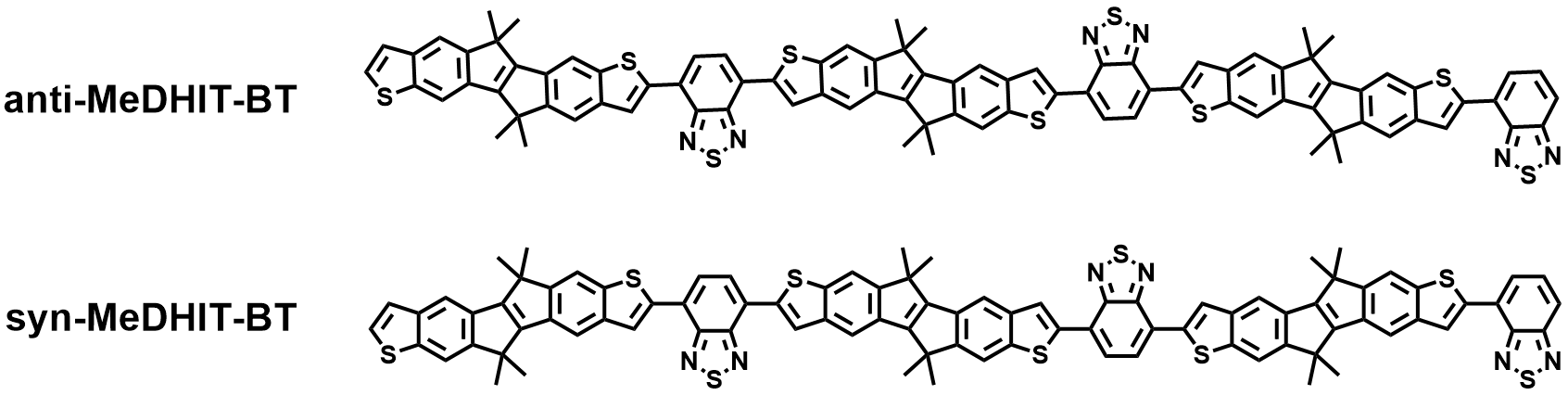


Figure S4. The chemical structure of trimers of the polymers.

Table S1. Calculated dihedral angles and energy levels for minimum energy geometries of trimers.

| Polymer | HOMO  (eV) | LUMO  (eV) | θ_1_  (°) | θ_2_  (°) |
| --- | --- | --- | --- | --- |
| anti-MeDHIT-BT | -5.17 | -2.97 | 1.1 | 6.5 |
| syn-MeDHIT-BT | -5.44 | -2.90 | 0.04 | 0 |


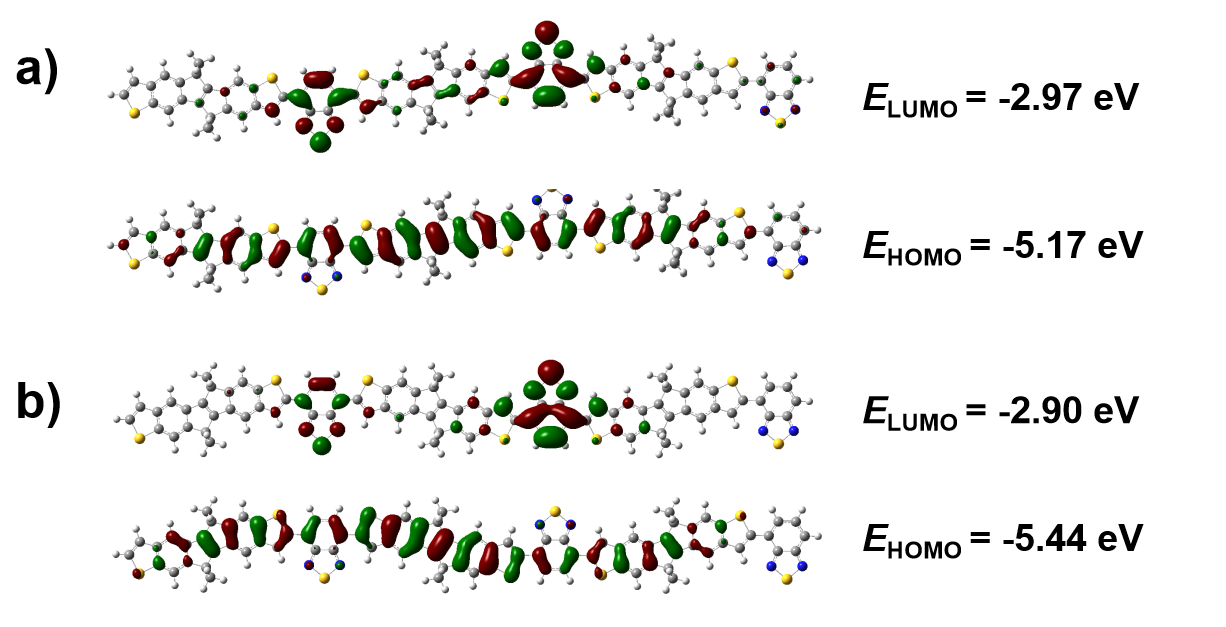


Figure S5. The frontier orbitals of a) anti-C16DHIT-BT and b) syn-C16DHIT-BT calculated by DFT.


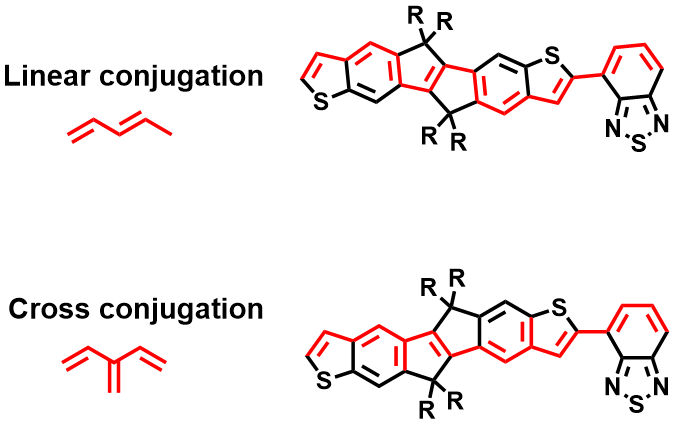


Figure S6. Schematic representation of the two connection modes in linear and cross-conjugated copolymers.


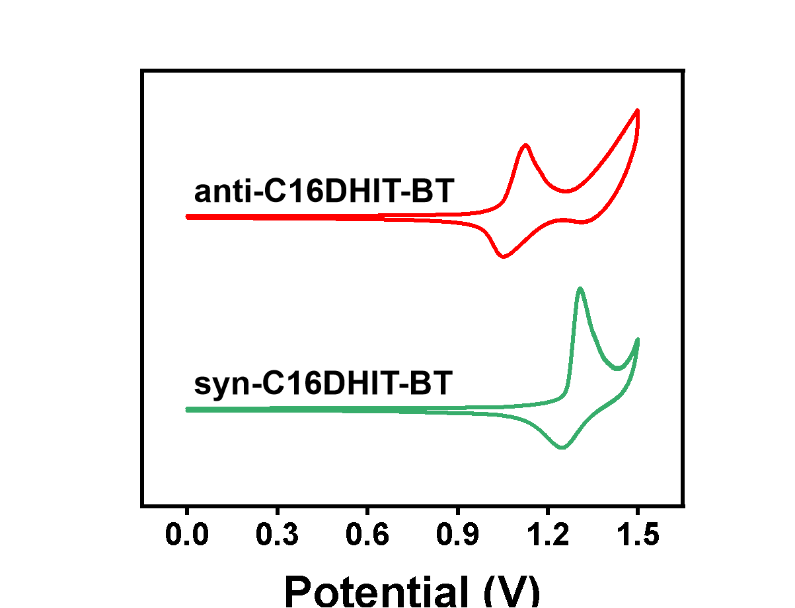


Figure S7. Film cyclic voltammograms (CV) of the polymers.

Table S2. Data summary of the edge-on oriented population of 2D GIWAXS patterns of the polymers.

| Polymer | IP | | | |  | OOP | | | |
| --- | --- | --- | --- | --- | --- | --- | --- | --- | --- |
|  | *q*  [Å^-1^] | *d*  [Å] | FWHM  [Å^-1^] | CL  [Å] |  | *q*  [Å^−1^] | *d*  [Å] | FWHM  [Å^−1^] | CL  [Å] |
| anti-C16DHIT-BT | 1.35 | 4.65 | 0.23 | 24.96 |  | 0.30 | 21.15 | 0.05 | 103.96 |
|  |  |  |  |  |  | 0.89 | 7.02 | 0.26 | 21.37 |
| syn-C16DHIT-BT | 1.37 | 4.58 | 0.20 | 28.71 |  | 0.29 | 21.71 | 0.05 | 106.28 |

Table S3. Data summary of the face-on oriented population of 2D GIWAXS patterns of the polymers.

| Polymer | OOP | | | |  | IP | | | |
| --- | --- | --- | --- | --- | --- | --- | --- | --- | --- |
|  | *q*  [Å^-1^] | *d*  [Å] | FWHM  [Å^-1^] | CL  [Å] |  | *q*  [Å^−1^] | *d*  [Å] | FWHM  [Å^−1^] | CL  [Å] |
| anti-C16DHIT-BT | 1.56 | 4.03 | 0.49 | 11.44 |  | 0.39 | 16.14 | 0.04 | 132.66 |
|  |  |  |  |  |  | 0.81 | 7.71 | 0.19 | 29.01 |
| syn-C16DHIT-BT | 1.57 | 4.00 | 0.42 | 13.33 |  | 0.39 | 15.94 | 0.04 | 152.23 |

Figure S7. ^1^H NMR spectrum (600 MHz, CDCl_3_) of **A2**.

Figure S8. ^13^C NMR spectrum (151 MHz, CDCl_3_) of **A2**.

Figure S9. ^19^F NMR spectrum (376 MHz, CDCl_3_) of **A2**.

Figure S10. ^1^H NMR spectrum (400 MHz, CDCl_3_) of **A3**.

Figure S11. ^13^C NMR spectrum (101 MHz, CDCl_3_) of **A3**.

Figure S12. ^19^F NMR spectrum (376 MHz, CDCl_3_) of **A3**.

Figure S13. ^1^H NMR spectrum (400 MHz, CDCl_3_) of **A4**.

Figure S14. ^13^C NMR spectrum (101 MHz, CDCl_3_) of **A4**.

Figure S15. ^19^F NMR spectrum (376 MHz, CDCl_3_) of **A4**.

Figure S16. ^1^H NMR spectrum (400 MHz, CDCl_3_) of **A5**.

Figure S17. ^13^C NMR spectrum (101 MHz, CDCl_3_) of **A5**.

Figure S18. ^19^F NMR spectrum (376 MHz, CDCl_3_) of **A5**.

Figure S19. ^1^H NMR spectrum (400 MHz, CDCl_3_) of **A6**.

Figure S20. ^13^C NMR spectrum (101 MHz, CDCl_3_) of **A6**.

Figure S21. ^19^F NMR spectrum (376 MHz, CDCl_3_) of **A6**.

Figure S22. ^1^H NMR spectrum (400 MHz, CDCl_3_) of **A7**.

Figure S23. ^13^C NMR spectrum (101 MHz, CDCl_3_) of **A7**.

Figure S24. ^19^F NMR spectrum (376 MHz, CDCl_3_) of **A7**.

Figure S25. ^1^H NMR spectrum (400 MHz, CDCl_3_) of anti-C16DHIT.

Figure S26. ^13^C NMR spectrum (101 MHz, CDCl_3_) of anti-C16DHIT.

Figure S27. ^1^H NMR spectrum (400 MHz, CDCl_3_) of anti-C16DHIT-Br.

Figure S28. ^13^C NMR spectrum (101 MHz, CDCl_3_) of anti-C16DHIT-Br.


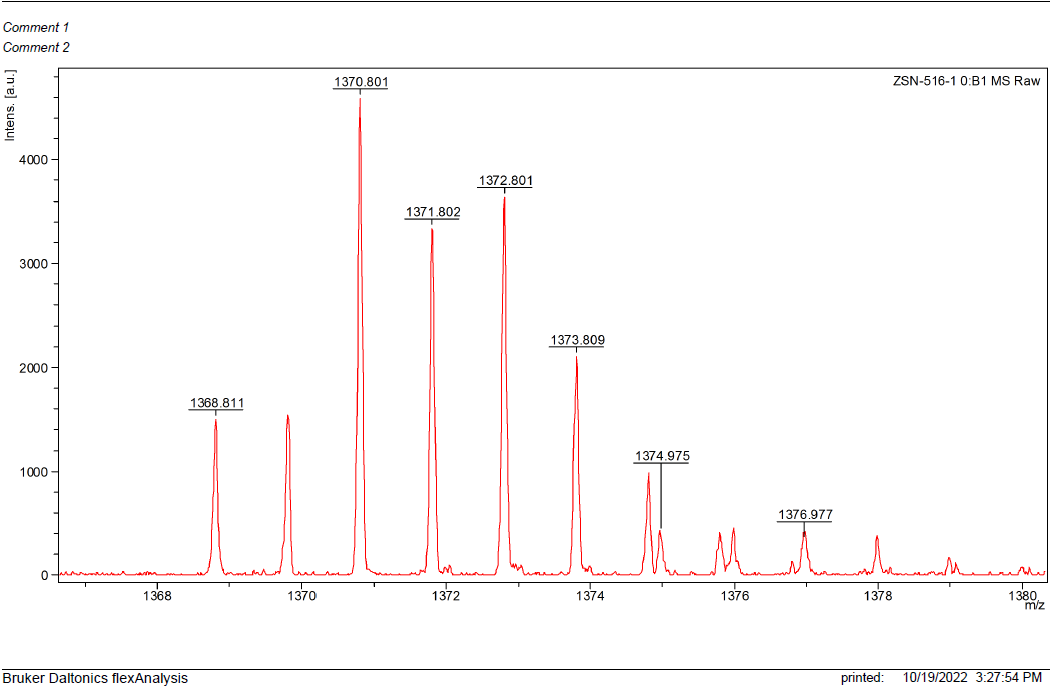


Figure S29. MALDI-TOF-MS of anti-C16DHIT-Br.

Figure S30. ^1^H NMR spectrum (600 MHz, CDCl_3_) of **B2**.

Figure S31. ^13^C NMR spectrum (151 MHz, CDCl_3_) of **B2**.

Figure S32. ^19^F NMR spectrum (565 MHz, CDCl_3_) of **B2**.

Figure S33. ^1^H NMR spectrum (600 MHz, CDCl_3_) of **B3**.

Figure S34. ^13^C NMR spectrum (151 MHz, CDCl_3_) of **B3**.

Figure S35. ^19^F NMR spectrum (565 MHz, CDCl_3_) of **B3**.


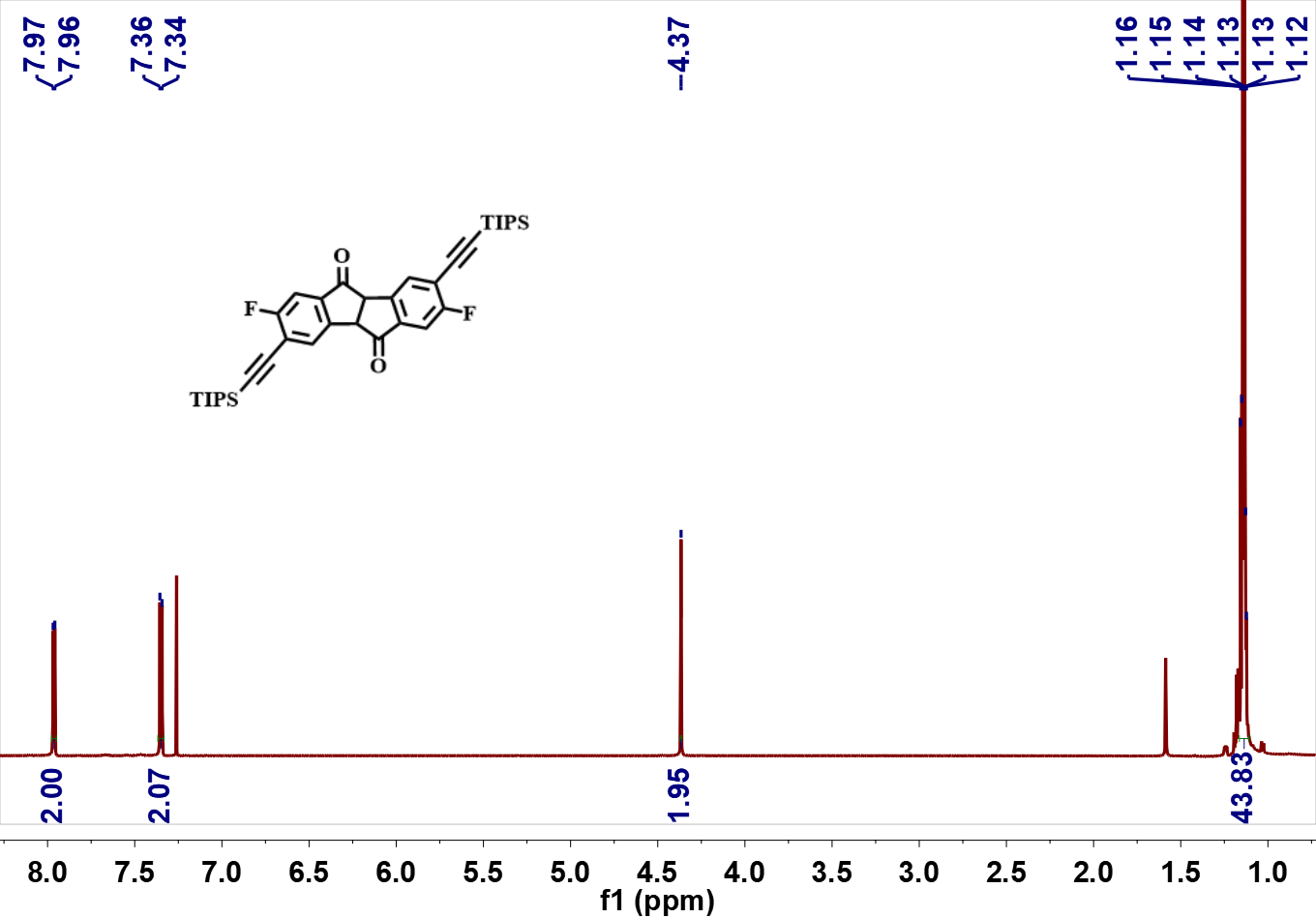


Figure S36. ^1^H NMR spectrum (600 MHz, CDCl_3_) of **B4**.

Figure S37. ^13^C NMR spectrum (151 MHz, CDCl_3_) of **B4**.

Figure S38. ^19^F NMR spectrum (565 MHz, CDCl_3_) of **B4**.

Figure S39. ^1^H NMR spectrum (600 MHz, CDCl_3_) of **B5**.

Figure S40. ^1^H NMR spectrum (151 MHz, CDCl_3_) of **B5**.

Figure S41. ^19^F NMR spectrum (565 MHz, CDCl_3_) of **B5**.

Figure S42. ^1^H NMR spectrum (600 MHz, CDCl_3_) of **B6**.

Figure S43. ^13^C NMR spectrum (151 MHz, CDCl_3_) of **B6**.

Figure S44. ^19^F NMR spectrum (565 MHz, CDCl_3_) of **B6**.

Figure S45. ^1^H NMR spectrum (600 MHz, CDCl_3_) of syn-C16DHIT.


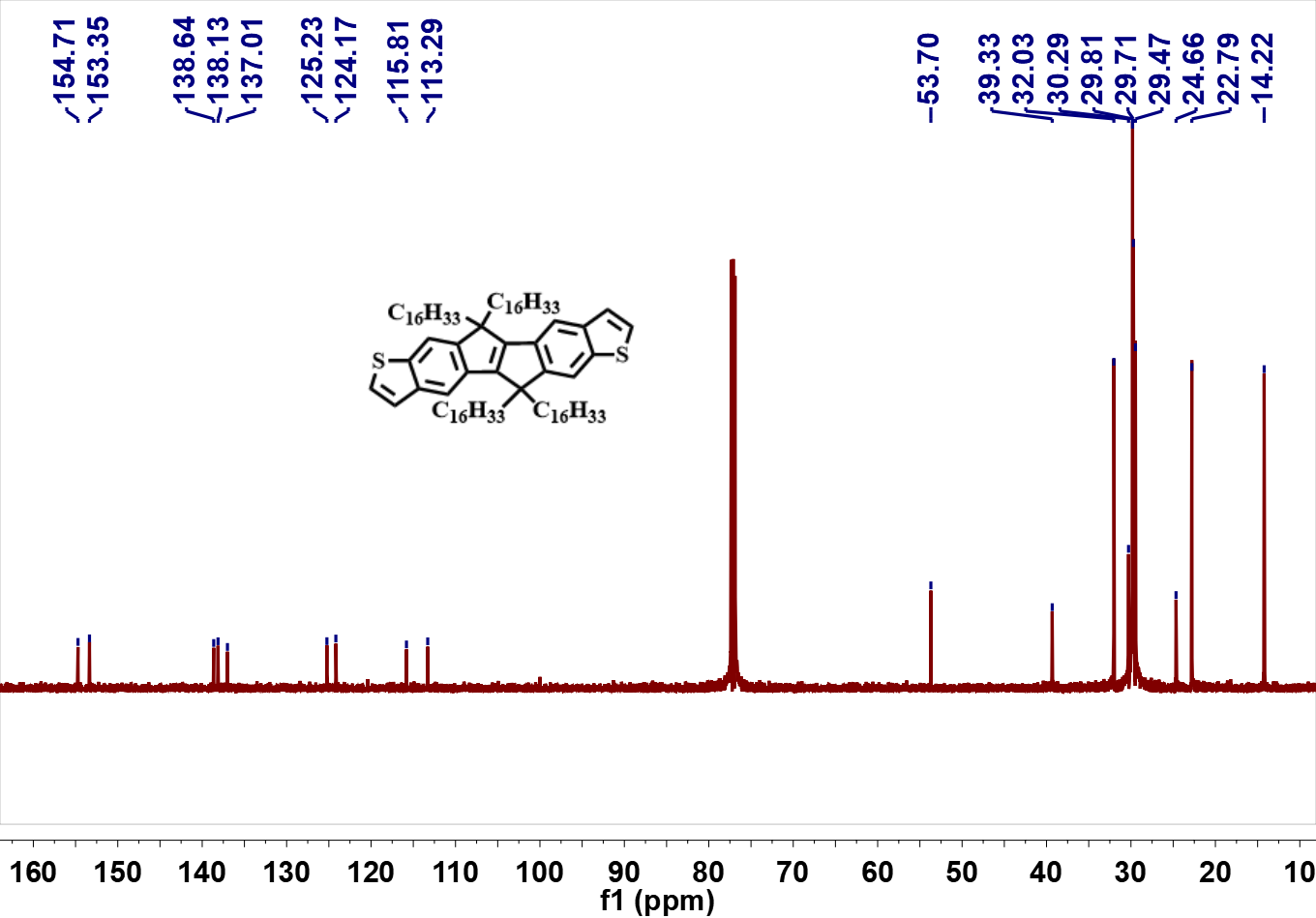


Figure S46. ^13^C NMR spectrum (151 MHz, CDCl_3_) of syn-C16DHIT.

Figure S47. ^1^H NMR spectrum (600 MHz, CDCl_3_) of syn-C16DHIT-Br.


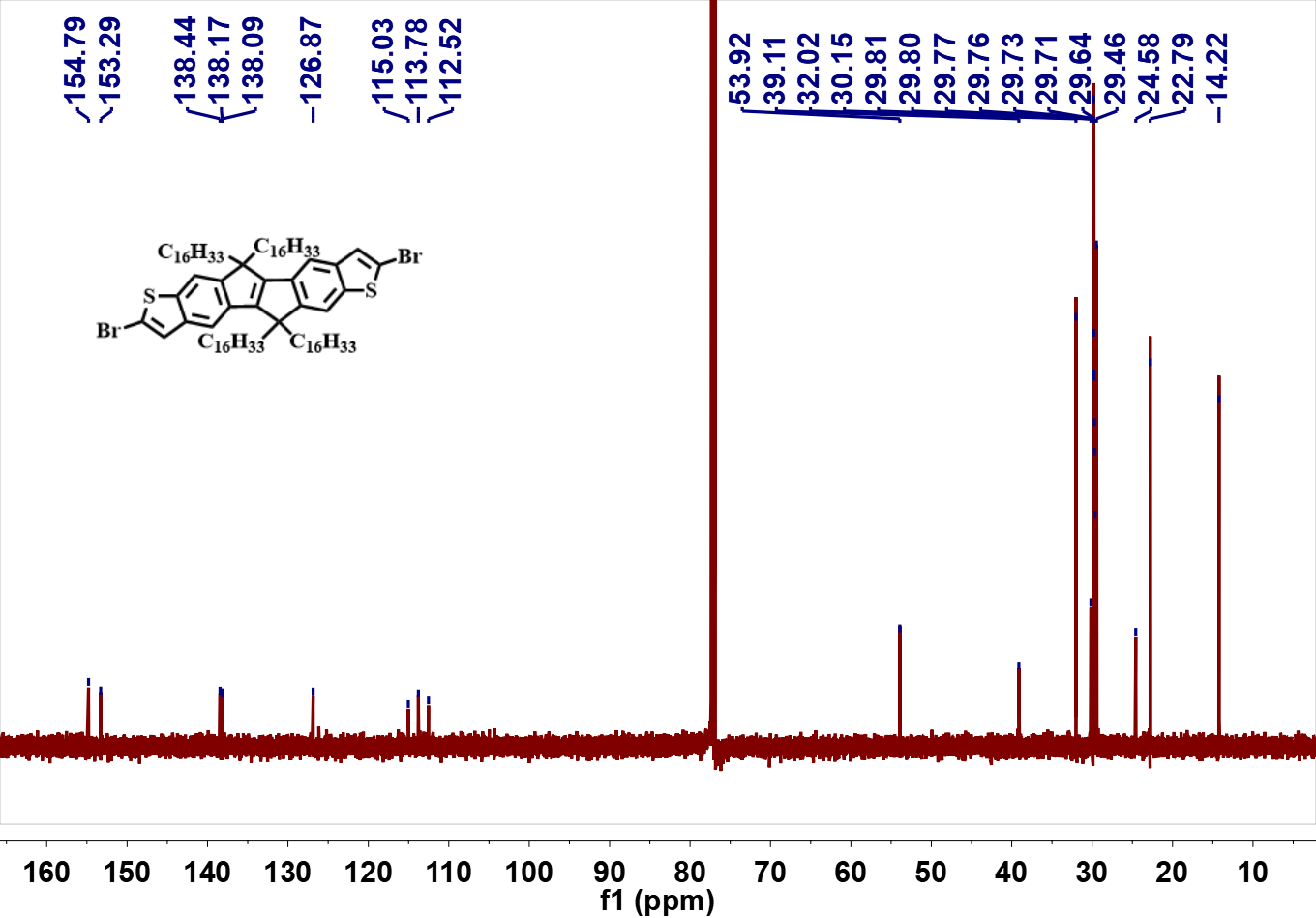
Figure S48. ^13^C NMR spectrum (151 MHz, CDCl_3_) of syn-C16DHIT-Br.


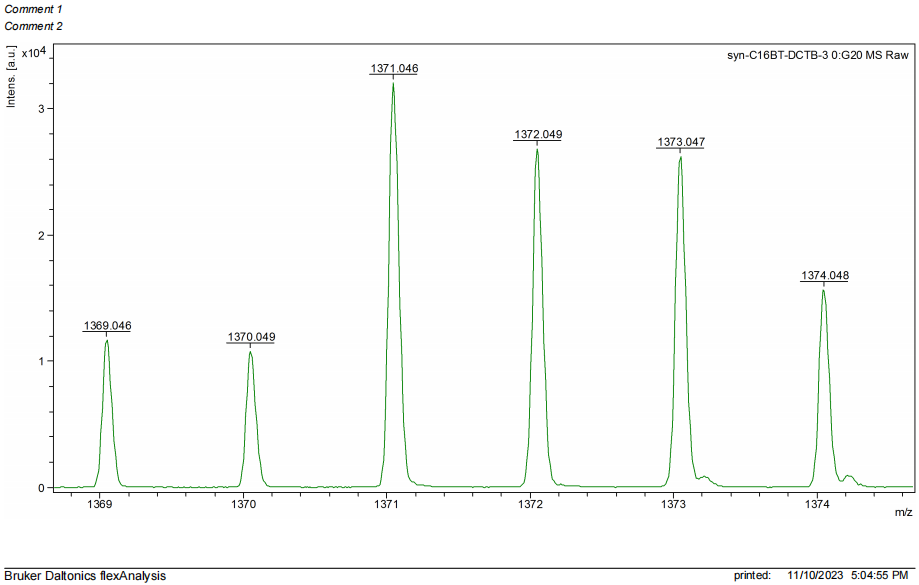


Figure S49. MALDI-TOF-MS of syn-C16DHIT-Br.

Figure S50. ^1^H NMR spectrum (600 MHz, CDCl_3_) of anti-C16DHIT-BT.

Figure S51. ^1^H NMR spectrum (600 MHz, CDCl_3_) of syn-C16DHIT-BT.
